# Molecular characterization of the murine Leydig cell lines TM3 and MLTC-1

**DOI:** 10.3389/fendo.2025.1715307

**Published:** 2025-12-16

**Authors:** Sarah K. Schröder-Lange, Katharina S. Hardt, Diandra T. Keller, Manuela Pinoé-Schmidt, Eva M. Buhl, Ralf Weiskirchen

**Affiliations:** 1 Institute of Molecular Pathobiochemistry, Experimental Gene Therapy and Clinical Chemistry (IFMPEGKC), Rheinisch-Westfälische Technische Hochschule (RWTH) University Hospital Aachen, Aachen, Germany; 2 Electron Microscopy Facility, Institute of Pathology, Rheinisch-Westfälische Technische Hochschule (RWTH) University Hospital Aachen, Aachen, Germany

**Keywords:** Leydig cell line(s), MLTC-1, TM3, cell authentication, STR profile, steroidogenesis, lipid droplets, electron microscopy

## Abstract

**Introduction:**

Leydig cells (LCs) are located in the interstitium of the testes and are characterized as steroid hormone-producing cells that are crucial for testicular function in maintaining spermatogenesis. Disruptions in their function, for instance due to genetic defects, endocrine disruptors, or age-related changes, often lead to hypogonadism and are among the main causes of male infertility. Therefore, reliable *in vitro* models are crucial for understanding the underlying signaling pathways, developing new therapeutic approaches and testing toxic substances that interfere with these processes. However, as primary LCs are naturally rare, require time-consuming isolation from animals and yield only small quantities, in theory, permanent LC lines offer a more practical and reproducible alternative.

**Methods:**

We used non-tumorigenic TM3 cells and tumorigenic MLTC-1 cells, two commercially available LCs, and subjected them to a comprehensive molecular and genetic comparative analysis.

**Results and discussion:**

Light microscopy and F-actin staining by Phalloidin revealed the typical polygonal LC shape of both cell lines growing in clusters with tight cell-cell contacts. Both cell lines contain endogenous lipid droplets (LDs) and synthesis was inducible with oleic acid. Transcriptomic analysis and RT-qPCR verified the expression of different steroidogenesis-associated LD genes including members of the PLIN family and lipolysis enzymes (e.g. *Pnpla2*, *Lipe*, *MgII*). In addition, we identified a comprehensive panel of steroidogenesis genes via RT-PCR, RT-qPCR and Western blot analysis in MLTC-1 cells, but these were not detectable in TM3 cells. In contrast to the originally described response to gonadotropins in TM3 cells, RNA sequencing revealed no *Lhcgr* expression and the cells are not responsive to stimulation with human chorionic gonadotropin (hCG). MLTC-1 cells are positive for *Lhcgr* and respond to hCG administration with strong StAR induction on the protein and mRNA level. Short tandem repeat (STR) profiling confirmed the authenticity of both cell lines used in our study. Taken together, comprehensive molecular characterization, which includes next generation sequencing (NGS)-based transcriptome analysis and STR profiling, is essential in confirming the validity of cell models and enhancing their significance in infertility research and endocrine toxicology.

## 1 Introduction

The interstitial Leydig cells (LCs) are the primary androgen-producing cells in the vertebrate testes (1). LCs are known for their significant steroidogenesis and the presence of relevant steroid receptors and enzymes. Cholesterol serves as the essential precursor for steroidogenesis synthesis in LCs. The conversion of cholesterol into testosterone by LCs is a multi-step process that involves cholesterol transporters, steroidogenic enzymes and various regulatory molecules (2). The enzymes necessary for steroidogenesis include the following (3): The steroidogenic acute regulatory protein (StAR) transports cholesterol into the mitochondrial membrane (rate-limiting step), where the cytochrome P450 cholesterol side-chain cleavage enzyme (CYP11A1) cleaves the cholesterol side chain to produce pregnenolone. 3β-hydroxysteroid dehydrogenase (3β-HSD) oxidizes and isomerizes pregnenolone to produce progesterone, then cytochrome P450 17α-hydroxylase/17,20-lyase (CYP17A1) hydroxylates and further cleaves it to androstenedione. Finally, the 7β-hydroxysteroid dehydrogenase type 3 (17β-HSD3) reduces androstenedione to testosterone. The testosterone produced controls spermatogenesis via the hypothalamic-pituitary-gonadal axis and influences secondary sexual characteristics. Disorders of LC function manifest clinically as hypogonadism and impair fertility and have also been discussed as a contributing factor to age-related diseases (2). Therefore, a detailed understanding of LC biology is highly relevant for both basic research and the development of new diagnostics and therapies for male infertility. Cell cultures serve as a fundamental *in vitro* model in biomedical research, enabling controlled investigation of toxicological, pharmacological, and genetic processes.

Despite intensive research, isolating primary LCs with high purity remains challenging (4–6). Various approaches have been established to successfully isolate and culture primary murine testicular cells (4, 5). This challenge is exacerbated by the small proportion of LCs in the interstitial cells, making up only around 5-10% (7). They constitute just 10.7% of the total volume of the mouse testicular parenchyma, with the remaining 89.3% consisting of the seminiferous tubules (8). While cells with high viability can be isolated, the limited quantity of cells is often a constraint. Only about 2-3×10^5^ LCs can be isolated per two testes in an optimized protocol (5, 9). Therefore, immortalized LC lines are crucial for investigating basic and applied research questions related to male-specific hormonal processes. These immortalized cell lines offer an infinite lifespan and low variability between experiments, serving as a vital tool in reproductive research (10, 11). They provide a controlled *in vitro* platform to study LC physiology, endocrine regulation and toxicological mechanisms without the immediate need for animal experimentation or human testicular specimens (10–13). However, there are growing debates on the utility of these immortalized LC lines (10–12). Unfortunately, the drawbacks of commercially available or specially generated cell lines have become increasingly apparent in recent decades. A significant issue in working with LCs is the phenomenon where the cells become insensitive to gonadotropins during cultivation or lose the expression of the receptor, thereby losing their ability to synthesize steroids, leading to truncated steroid pathways (12, 14, 15).

To the best of our knowledge, no human LC line is currently available. However, there are some rodent LC lines, but their functional properties, hormone responses and molecular characteristics are either inconsistent or partially unknown (10–12). The only non-tumorigenic murine testicular cell line believed to stem from LC is the TM3, which was isolated and established by Mather *et al*. in 1980 (16). TM3 cells are derived from 11 to 13 days-old immature BALB/c mice (16). The cell line was characterized by the receptor for human chorionic gonadotropin (hCG)/luteinizing hormone (LH) (encoded by the *Lhcgr* gene) and responsiveness to LH stimulation with increased cAMP levels (16, 17). However, within the last 40 years, researchers have failed to reproduce these findings (10). TM3 cells are distributed by the American Type Culture Collection (ATCC) as CRL-1714^™^ and have been used in almost 400 studies according to the PubMed database as of end of September 2025. One of the most widely studied murine tumorigenic LCs is the MLTC-1 cell line. This cell line was established from a transplanted LC tumor (M5480P) in C57B1/6 mice by Rebois *et al*. in 1982 (18). Despite their tumorigenic origin, MLTC-1 cells have been described as hormone-responsive, with an increase in cAMP levels observed upon stimulation with hCG or LH (18, 19). However, it became apparent that MLTC-1 produced more progesterone in response to hCG stimuli and only small amounts of testosterone, the final product of steroid hormone synthesis (10, 18, 20). Nowadays, MLTC-1 cells are commercially available via ATCC (CRL-2065™) and according to the PubMed database, have been used in over 150 studies. Recently, genetic characterization studies found that these cells share an approximately 40% similarity to human LC tumors, but also other types of testicular tumors (21).

Despite being used for over 40 years, there is a lack of comprehensive molecular and genetic characterization of these important tools in endocrine research. Insufficient characterization of the cell lines used, along with phenotypic drift, lack of reproducibility of original findings, and potential cell line contaminations, poses a growing challenge to experimental validity (22, 23). When utilizing permanent cell lines, it is crucial to address the issue of authentication. Cell lines are widely accepted tools for studying normal and pathological cell functions in biomedical research, yet authentication is often overlooked (23, 24). This typically involves transcriptomic analysis of the cells and determining their short tandem repeat (STR) profile (22, 25).

In light of these challenges, the present study aims to systematically characterize the two commonly used immortal LC lines, TM3 and MLTC-1. We aimed to provide a thorough genetic characterization of these cells using STR profiling to confirm their identity and stability. To gain insights into the gene expression patterns, we conducted bulk mRNA-seq via next-generation sequencing (NGS). Additionally, we performed morphological analysis of the LC lines using Phalloidin staining, as well as microscopic and ultrastructural analysis. To assess the functional properties of these cells, we conducted stimulation experiments using hCG. Our study offers important insights into the molecular characterization of these cell lines that will be valuable for future endocrine research.

## 2 Material and methods

### 2.1 Cell culture and light microscopy

The murine LC lines MLTC-1 (#ATCC-CRL-2065) and TM3 (#ATCC-CRL-1714) that were used in this study were obtained from the ATCC via LCG Standards (Wesel, Germany). MLTC-1 cells were cultured in ATCC-formulated RPMI-1640 Medium (#A1049101, Gibco™, Thermo Fisher Scientific, Schwerte, Germany) supplemented with 10% fetal bovine serum (FBS, #F7524, Sigma-Aldrich) and 1× penicillin-streptomycin antibiotic solution (P0781-100ML, Sigma Life Science, Lonza, Cologne, Germany). The LC line TM3 was cultured in Dulbecco’s Modified Eagle Medium: Nutrient Mixture F-12 (DMEM/F-12, #11330057, Gibco™, Thermo Fisher Scientific, Schwerte, Germany) supplemented with 5% horse serum (#16050122, Gibco™, Thermo Fisher Scientific, Schwerte, Germany), 2.5% FBS and 1× penicillin-streptomycin antibiotic solution. The medium of the cell lines was changed every two days. Both cell lines were kept in 10 cm dishes in a humidified incubator at 37°C with 5% CO_2_ and subcultured every three to four days in a ratio of 1:3 (MLTC-1) or 1:30 (TM3) using Accutase^®^ solution (A6964-100ML, Sigma-Aldrich).

Cellular morphology was routinely monitored using light microscopy. To assess the morphology of the cell lines, bright-field and phase-contrast images were taken at different densities using a Leica EC3 digital camera connected to a Leica DM IL LED microscope fitted with the Leica Application Suite software (version 3.4.0, Leica Microsystems GmbH, Wetzlar, Germany).

### 2.2 Activation of steroidogenesis

The activation of steroidogenesis was assessed by stimulating murine LC lines with hCG. For this, 450,000 MLTC-1 or 200,000 TM3 cells were seeded in 6-well culture plates containing supplemented standard medium (refer to Section 2.1). After 24 hours, the medium was switched to standard medium without serum for an additional 24 hours. The cells were then stimulated with different concentrations of hCG (reconstituted with dH_2_O to a concentration of 1,000 IU/mL, #CG5-1VL, Sigma-Aldrich) in serum-free standard medium with all the necessary supplements for the indicated times (6 and 24 hours). Staining for 3β-HSD activity, protein or RNA analysis was conducted as described below.

### 2.3 F-actin staining using phalloidin

F-actin stress fibers were visualized using a rhodamine phalloidin probe (#R415, Invitrogen™, Thermo Fisher Scientific). For this, 35,000 MLTC-1 or 25,000 TM3 cells were seeded per well in four-chamber polystyrene vessel tissue culture-treated glass slides (#354114, Falcon^®^, Corning Life Science, Tewksbury, MA, USA) and cultured to 80% confluence. A detailed experimental procedure of the phalloidin staining including all washing steps with 1× PBS, has been previously described (26). In brief, fixation of the cells was done in 4.0% paraformaldehyde (buffered with phosphoric acid to pH 7.4) for 20 minutes in the dark, followed by permabilization with precooled 0.1% sodium-citrate/0.1% Triton X-100 in PBS for 3 minutes on ice. Non-specific binding sites were blocked with 0.5% bovine serum albumin (BSA) in PBS for 1 hour at room temperature. All subsequent steps were carried out in the dark. F-actin stress fibers were stained with 1× phalloidin rhodamine solution for 20 min, followed by nuclear counterstaining with 4′,6-diamidino-2-phenylindole (DAPI, #D1306, Thermo Fisher Scientific) for 30 minutes. Finally, the slides were mounted in aqueous VECTASHIELD^®^ Antifade Mounting Medium (#H-1000-10, Vector Laboratories, Burlingame, CA, USA) and stored in the dark at 4 °C until analysis. Fluorescence images were captured with a Nikon Eclipse E80i microscope and NIS-Elements Vis software (Version 3.22.01, Nikon Instruments, Düsseldorf, Germany).

### 2.4 Staining for 3β-HSD activity

The 3β-HSD activity can be visualized as previously described to distinguish LCs in a testicular population (27, 28). Modification of the protocol has been adapted for the characterization of primary mouse LCs in culture (5, 6). These protocols were used to stain the 3β-HSD activity of TM3 and MLTC-1 cells. For this, cells were cultured on four-chamber slides as described before. Both cell lines were treated with or without 5 IU/mL hCG for 6 hours to induce steroidogenesis (see Section 2.2). After fixation with 4.0% paraformaldehyde for 20 minutes in the dark, cells were washed twice in PBS and dried for 1 hour at 37 °C. For staining, solution A and solution B were required. Solution A consists of 1 mg nitrotetrazolium blue chloride (NTB, #N6876-100MG, Sigma-Aldrich) and 0.6 mg dehydroepiandrosterone (DHEA, #252805-10-MG, Sigma-Aldrich) in 0.6 ml DMSO. Solution B is made of 5 mg β-nicotinamide adenine dinucleotide hydrate (β-NAD, #N1511-250MG, Sigma-Aldrich) in PBS. For staining, 0.3 mL of solution A and 4.75 mL of solution B were freshly mixed and applied for 1 hour on the dried cells at 37 °C to stain β-HSD activity. After washing in PBS, slides with cells were mounted in VECTASHIELD^®^ Antifade Mounting medium and observed under the Nikon Eclipse E80i microscope (with NIS-Elements Vis software).

### 2.5 Lipid droplet staining

The endogenous amount of LDs was analyzed in MLTC-1 and TM3 cells using fluorescence and histological staining protocols. Oleic acid (OA) was used to stimulate LD production as a positive control in both LC lines. For LD assessment, 35,000 MLTC-1 or 25,000 TM3 cells were seeded per well in four-chamber polystyrene vessel tissue culture-treated glass slides. The next day, the medium was changed to serum-free medium. After 24 h, cells were treated with or without 0.25 mM OA (#O3008-5ML, Sigma-Aldrich) for an additional 24 hours.

Fluorescence detection of LDs was performed in the dark and cells were fixed and permabilzed as described in Section 2.2. Instead of the Phalloidin Rhodamine probe, the small bioactive molecule 4,4-difluoro-1,3,5,7,8-pentamethyl-4-bora-3a,4a-diaza-s-indacene (BODIPY™ 493/503, #D3922, Invitrogen™, Thermo Fisher Scientific) was used. A stock solution of 5 mM was prepared in DMSO and stored in the dark at -20°C for long term. Cells were stained with 6 µM BODIPY™ 493/503 in PBS for 60 minutes with gentle agitation at room temperature, and then the nuclei were counterstained with DAPI. The mounting and storage of the samples, and the subsequent analysis, were carried out as described in Section 2.2.

Oil Red O (ORO) can be used to stain and visualize the localization of LDs in cultured cells. For ORO staining, the medium was removed from the chamber glass slides and cells were fixed in 4% PFA for 20 minutes in the dark at room temperature. Next, the PFA solution was discarded, cells were washed with dH_2_O twice, and incubated in a 60% isopropanol solution for 5 minutes. A 0.5% ORO stock solution (#O0625, Sigma Aldrich) was prepared in 100% isopropanol and stored at room temperature. LD staining was done in a 0.3% ORO solution in dH_2_O for 25 minutes. After additional washing steps, nuclei were stained using Mayer’s Hematoxylin (Lilies’s Modification, #S3309, Agilent Technologies, Inc., Santa Clara, CA, USA) diluted 1:3 in dH_2_O for 5 min. Afterwards, the slides were washed under running tap water for 10 minutes. Finally, the ORO-stained cells were mounted in aqueous VECTASHIELD^®^ Antifade Mounting Medium and stored in the dark at 4°C until analysis. Images were captured with a Nikon Eclipse E80i microscope and NIS-Elements Vis software.

### 2.6 Protein analysis by Western blot

For endogenous protein analysis, 450,000 MLTC-1 or 200,000 TM3 cells were seeded in 6-well culture plates and cultured for three days in standard medium with supplements (see Section 2.1). After washing the cells with cold 1× PBS twice on ice, cells were harvested in RIPA buffer containing 50 mM Tris-HCl (pH 7.4), 150 mM NaCl, 1% (*w*/*v*) NP-40, 0.1% (*w*/*v*) sodium dodecyl sulfate, 0.5% (*w*/*v*) deoxycholic acid sodium salt supplemented with a cocktail of cOmplete™ protease inhibitor (#11697498001, Roche Diagnostics, Mannheim, Germany) and phosphatase inhibitor (#P5726-1ML, Sigma-Aldrich). Protein extracts from murine testis (adult wild type mice) collected in 2023 and stored at -80°C served as a control (29). The collection of these tissues was approved by the internal Institutional Review Board for studies involving animals (permit number: 40138A4; date of approval 8 September 2021). As described in Kessel *et al.*, 2023, these tissue samples were homogenized in RIPA buffer using a MM400 mixer mill (Retsch GmbH, Haan, Germany) (29).

The protein content of each sample was quantified by DC protein assay (#500-0116, Bio-Rad Laboratories, Düsseldorf, Germany). Equal amounts of proteins (40 µg) were mixed with dithiothreitol as a reducing agent and Nu-PAGE™ LDS electrophoresis sample buffer (#NP0008, Thermo Fisher Scientific) and heated at 80°C for 10 min before separation using sodium dodecyl sulfate-polyacrylamide gel electrophoresis (SDS-PAGE). For this, 4–12% Bis-Tris gradient gels (#NP0335BOX or #WG1402BOX, Thermo Fisher Scientific) and 2-(*N*-morpholino) ethanesulfonic acid (MES) running buffer were used. Proteins were transferred onto a nitrocellulose membrane (#GE10600002, 0.45 μm, Merck, Darmstadt, Germany) and equal protein loading and successful transfer were confirmed by Ponceau S stain. The membranes were blocked with 5% (*w*/*v*) non-fat milk powder in Tris-buffered saline with Tween^®^ 20. Different primary antibodies were incubated overnight at 4°C with gentle agitation. For visualization, secondary antibodies coupled to horseradish peroxidase with SuperSignal™ West Dura Extended Duration Substrate (#34076, Thermo Fisher Scientific) were used. Details of all primary and secondary antibodies can be found in **Supplementary Table 1**.

### 2.7 RNA extraction, real time-PCR and real-time quantitative PCR

For RNA analysis, 450,000 MLTC-1 or 200,000 TM3 cells were seeded in 6-well culture plates and cultured for three days in standard medium with supplements (see section 2). As a control, testis tissue from a 3-month-old mouse collected in 2023 was used (29). Testis tissue samples were homogenized using a MM400 mixer mill (Retsch GmbH). The PureLink™ RNA Mini-Kit (#12183018A, Invitrogen™, Thermo Fisher Scientific) including DNase digestion (#12185010, Invitrogen™, Thermo Fisher Scientific) was used to extract and purify total RNA from murine LC lines and testis tissue. Detailed protocols for RNA extraction and purification, as well as complementary DNA (cDNA) synthesis (from one µg of RNA), reverse transcription, quantitative real-time PCR (RT-qPCR) and conventional RT-PCR were previously published (29–31). A list of all primers included in this study is given in **Supplementary Table 2**.

### 2.8 Electron microscopy

For ultrastructural analysis, MLTC-1 and TM3 cells were seeded on 10 cm dishes and treated as described in Section 2.2. The cell lines were treated with either 0.25 mM OA, 5 IU/mL hCG or left untreated in serum-free standard medium with all supplements. The cells were fixed in a freshly prepared solution of 3% glutaraldehyde in a 0.1 M phosphate buffer at room temperature, followed by post-fixation in a 1% osmium tetroxide solution. After dehydration in graded ethanol solutions, the samples were embedded in epoxy resin. Ultrathin sections (approximately 90–100 nm thickness) were prepared using an ultramicrotome and placed on copper grids. The sections were then stained with 0.5% uranyl acetate and 1% lead citrate and examined with a Zeiss Leo 906 transmission electron micro-scope (Carl Zeiss AG, Oberkochen, Germany) operating at 60 kV. High-resolution micro-graphs taken at magnifications ranging from 2,784× to 27,800× enabled a detailed evaluation of organelle preservation, including the structure of LDs, endoplasmic reticulum (ER), and other organelles characteristic of LCs.

### 2.9 Short tandem repeat profiling

STR profiling of the murine LC lines TM3 and MLTC-1 was conducted at IDEXX Laboratories in Kornwestheim, Germany. The CellCheck™ Mouse 19 STR Profile and Interspecies Contamination Test were utilized for this analysis. TM3 and MLTC-1 cell pellets were submitted to IDEXX for testing. The CellCheck™ Mouse 19 panel, designed to target 19 mouse-specific STR loci, was employed to confirm the genetic authenticity of the cell lines and identify any potential cross-species contamination. The results were presented in an STR report, which confirmed the murine origin of both cell lines and provided a distinct and expected genetic profile for each.

### 2.10 Mycoplasma testing

Regular mycoplasma testing was conducted in our laboratory using supernatant from cultured cells that were at least 90% confluent and grown for 3 days. Samples were directly analyzed with the Venor®GeM OneStep kit for conventional PCR (#11-8050, Minerva Biolabs GmbH, Berlin, Germany) as previously described (23, 32). The resulting amplicons were visualized on a 2% standard agarose gel, showing that both cell lines were negative for contamination with *Mycoplasma* spp. (**Supplementary Figure 1**).

### 2.11 Evaluation and statistical analysis

In this study, all calculations were performed in Excel v16 (Microsoft Corporation, Redmond, WA, USA). Unless otherwise stated, each experiment was independently conducted three times (n=3). In RT-qPCR, each biological replicate was analyzed in triplicate to ensure consistent and reproducible data. The mRNA expression of targets was quantified and displayed relative to the housekeeping gene ribosomal protein S6 (*Rps6*). For evaluation, the 2^-ΔΔCt^ method was applied (33). The expression of targets in MLTC-1 was compared to that in TM3 cells. In the stimulation experiments, the treatments were compared to the control condition.

Statistical analysis was performed with GraphPad Prism v.8.0 (GraphPad Software, Inc., La Jolla, CA). Gaussian distribution was tested with Shapiro-Wilk Tests. Student’s *t*-test was applied if normality could be assumed; otherwise, a non-parametric Mann-Whitney Test was applied. All data in this study are shown as mean ± standard deviation (SD). Statistical significance between groups was assumed when probability values were below 0.05 (*p* < 0.05). Significant differences are indicated by asterisks: * *p* < 0.05, ** *p* < 0.01, *** *p* < 0.001.

## 3 Results

### 3.1 Morphology of TM3 and MLTC-1 Leydig cells

**Figure 1:**
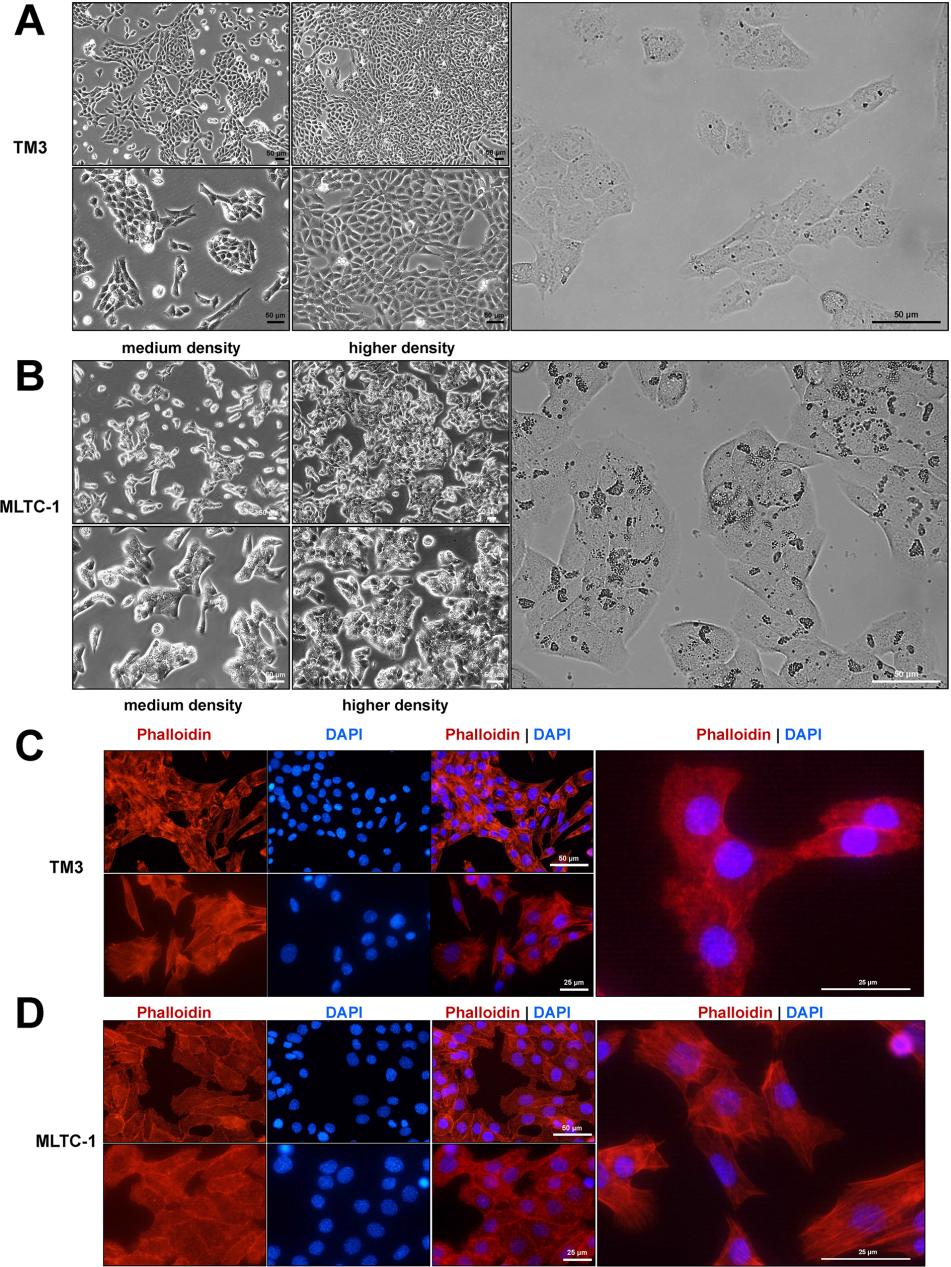
Morphology of murine TM-3 and MLTC-1 Leydig cells (LCs). Light microscopy images of cultured TM-3 **(A)** and MLTC-1 **(B)** cells at medium and higher densities display their polygonal shape as they grow in cell clusters. Scale bars equal 50 µm at 100×, 200× or 400× magnification. Refractive inclusions are visible at 400× magnification. **(C, D)** The F-actin cytoskeleton of LC lines was labeled with Rhodamine-Phalloidin (red) and nuclear counterstaining was done using DAPI (blue). Nuclei of both LC lines demonstrate prominent nucleoli. Images were captured at magnifications of 400× and 600×. Scale bars are shown at different magnifications (50 µm and 25 µm).

Initially, non-tumorigenic TM3 and tumorigenic MLTC-1 cells underwent a comparative morphological analysis. As isolated primary LCs, both cell lines exhibited adherent growth behavior in monolayers and an epithelial morphology (**Figure 1**). Light microscopy revealed that TM3 and MLTC-1 cells were polygonal and versatile. TM3 cells grew in clusters at low densities and formed an almost confluent monolayer when grown at high densities. In contrast, MLTC-1 cells continued to grow in clusters/islands and never became fully dense (**Figures 1A, B**). Under high magnification, some refractive inclusions were visible in TM3, while many were present in MLTC-1. As these inclusions in LCs are typically neutral lipids, the next step of our investigation focused on the intracellular lipid distribution between TM3 and MLTC-1 cells (see Section 3.3). These LDs could be distributed along the actin cytoskeleton within the cell. The actin cytoskeleton plays an important role in LCs in terms of cell shape, stability, and physiological processes such as migration or steroid production (34). F-actin stress fibers were visualized in TM3 and MLTC-1 cells by Phalloidin-Rhodamine staining (**Figures 1C, D**) and counterstaining with DAPI revealed large, oval-shaped nuclei with characteristic nucleoli. Both cell lines displayed strong cell-cell contacts to maintain a polygonal shape and grow in monolayers. Besides cell-cell contacts, cellular communication is important in testicular cells to regulate spermatogenesis (35).

Our transcriptomic analysis of both cell lines revealed high abundance of different markers for cellular contacts or communication including gap junction protein alpha 1 (*Gja*), catenin beta 1 and delta 1 (*Ctnnb1, Ctnnd1*), integrin alpha 5 (*Itga5*), integrin beta 1 (*Itgb1*), vinculin (*Vcl*), paxillin (*Pxn*), protein tyrosine kinase 2 (*Ptk2*) or vimentin (*Vim*) in TM3 and MLTC-1 cells (**Supplementary Table 3**). Furthermore, components of extracellular matrix proteins were found in the cell lines such as different collagens (such as *Col1a1*, *Col3a1*, *Col4a1*), fibronectin (*Fn1*) or adhesive glycoproteins such as laminins (*Lama1*, *Lama4*, *Lamb1*, *Lamb2*, *Lamc1*).

Although light microscopy and F-actin staining provided an initial overview of cellular morphology, the subcellular organization of these cells remains beyond optical resolution. To visualize the different organelles, including the structure of the ER and the characteristic mitochondria of steroidogenic cells, as well as their spatial relationships in detail, we performed ultrastructural analyses by transmission electron microscopy (TEM).

**Figure 2:**
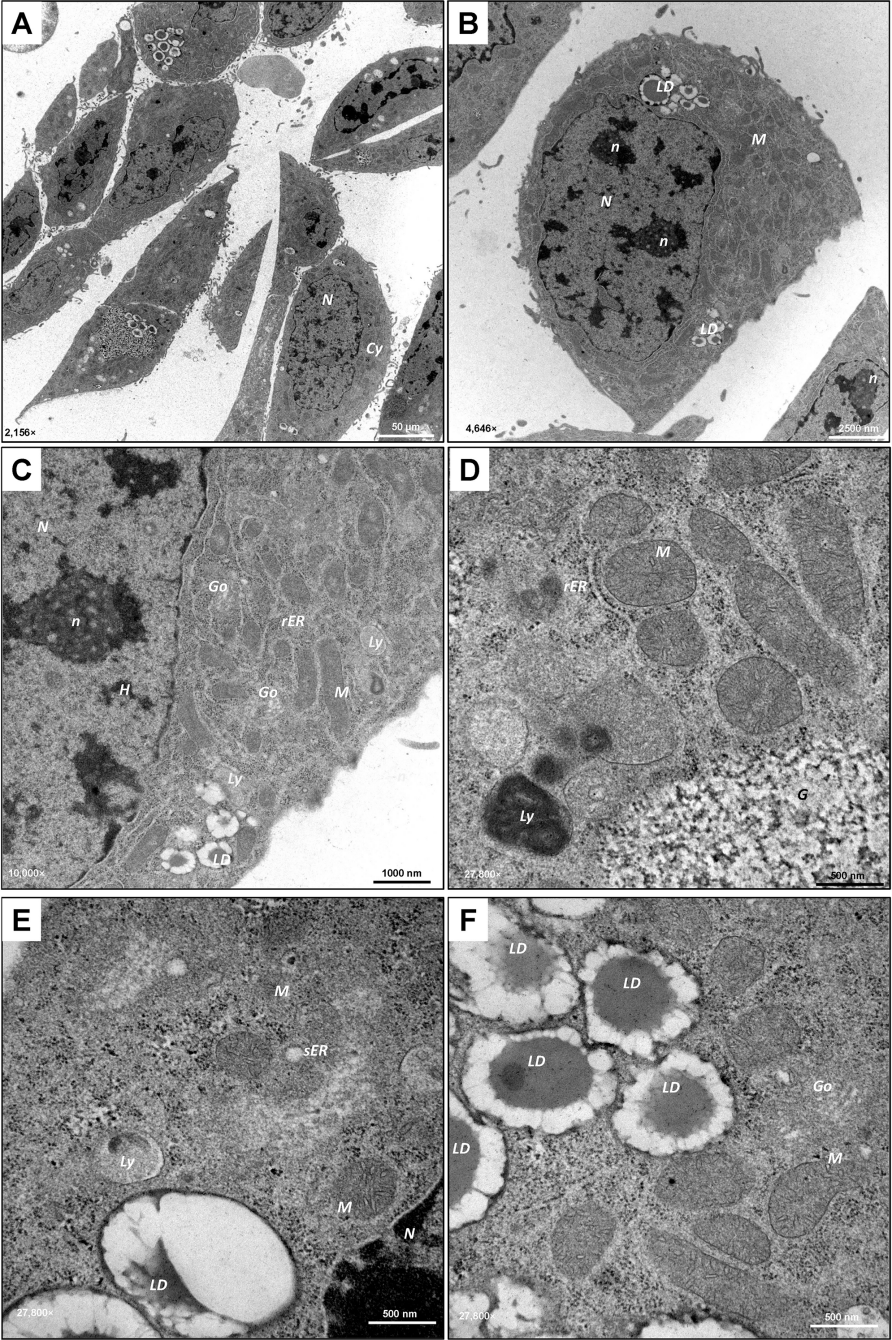
Ultrastructural analysis of MLTC-1 cells. **(A–F)** Transmission electron microscopy (TEM) demonstrates the heterogeneous appearance (polygonal, rounded or elongated shape) of the MLTC-1 LC line. The cells contain a prominent large nucleus (*N*) with electron-dense heterochromatin (*H*) and visible nucleoli (*n*). MLTC-1 cells exhibit many mitochondria (*M*) with tubular cristae,visible endoplasmic reticulum (*ER*) of smooth (*sER*) or rough (*rER*) type and Golgi apparatus (*Go*). Lytic vesicles (*Ly*) of various sizes and glycogen (*G*) can be found in the cytoplasm (*Cy*) of the cells. MLTC-1 cells contain several lipid droplets (*LDs*) which appear as light, circular areas with an electron-dense dark frame. TEM images were captured at 2,156× **(A)**, 4,646× **(B)**, 10,000× **(D)** and 27,800× **(D, F)** with scale bars representing 50 µm, 2500 nm, 1000 nm, or 500 nm.

**Figure 3:**
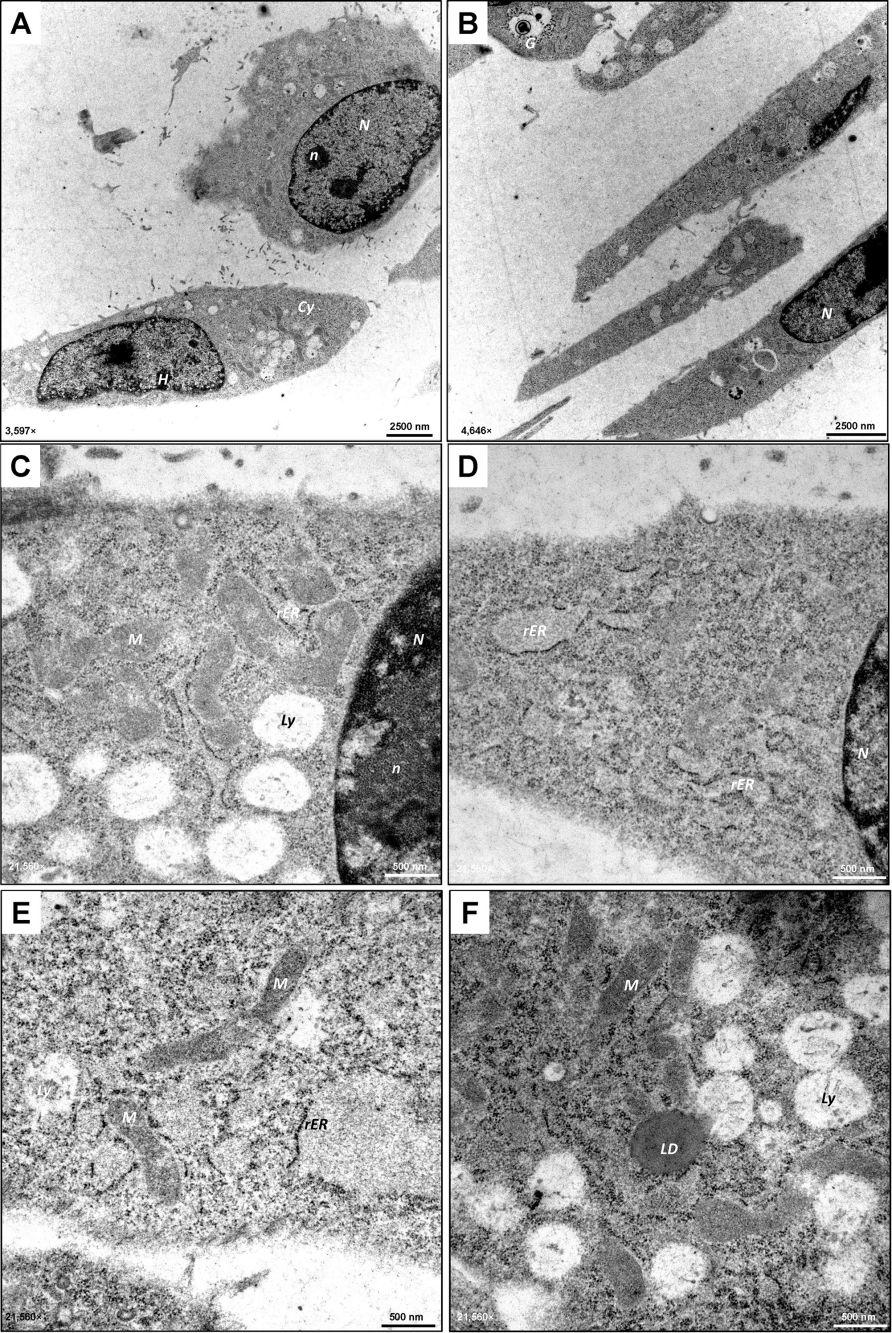
Ultrastructural analysis of TM3 cells. **(A–F)** TEM demonstrates the heterogeneous appearance (polygonal, rounded or elongated shape) of the TM3 Leydig cell line. The cells contain a prominent nucleus (*N*) with electron-dense heterochromatin (*H*) and visible nucleoli (*n*). TM3 cells exhibit numerous mitochondria (*M*) of tubular type with cristae and visible endoplasmic reticulum (*ER*) of smooth (*sER*) or rough (*rER*) type. Lytic vesicles (*Ly*) of various sizes and glycogen (*G*) can be found in the cytoplasm (*Cy*) of the cells. TM3 cells also contain some lipid droplets (*LD*) which appear as dark, circular areas with electron-dense dark frames. TEM images were captured at 3,597× **(A)**, 4,646× **(B)**, 21,560× **(C–F)** with scale bars representing 2500 nm or 500 nm.

Consistent with light microscopy imaging, TEM revealed a heterogeneous appearance, with some MLTC-1 cells having a polygonal, rounded shape and others being more elongated (**Figure 2A**). MLTC-1 cells are characterized by their large, prominent lobed nucleus (36) containing condensed heterochromatin (**Figures 2B, C**). This appears as dark, irregular electron-dense regions within the nucleus, contrasting with non-condensed euchromatin, which is abundant in the LCs, indicating active metabolism. In addition, the cell nuclei have clearly defined light grey areas known as nucleoli (**Figures 2B, C**). In the cytoplasma of the cells, the mitochondria appear as oval-shaped, slightly elongated, electron-dense structures. They mainly show the tubular type with cristae (**Figures 2D–F**) which is common for steroidogenic cells (37). In addition to the rough endoplasmic reticulum (rER), which exhibits flat, cistern-like membranes with ribosomes sitting on the surface (**Figure 2D**), the cell lines have several regions with smooth endoplasmic reticulum (sER), which appears as bright, tubular areas without ribosomes (**Figure 2E**). The ER is located in close vicinity to the abundant mitochondria in the cytoplasmic body of the cells (**Figure 2D**). Furthermore, there are lytic vesicles, potentially lysosomes (**Figures 2C–E**). Additionally, glycogen was detected in the cytoplasm, appearing as electron-dense granules in TEM (**Figure 2A, D**). As initially suspected through light microscopy, the presence of LDs was confirmed by TEM. MLTC-1 cells produce large LDs (**Figure 2F**) localized perinuclear, which are fundamental for appropriate steroidogenesis (38). In contrast to MLTC-1 cells, the pre-reproductive TM3 LC line demonstrates a more stretched and elongated appearance in TEM (**Figures 3A, B**). TM3 cells show a great amount of lipolytic vesicles and a strong presence of rER and sER (**Figures 3A, C–F**). The lumina are enlarged, which may result from accumulation of proteins inside. TM3 cells tend to produce smaller LDs compared with MLTC-1 cells. Overall, the morphological appearance confirms the LC-like origin of TM-3 and MLTC-1 cells.

### 3.2 Short tandem repeat profiling of TM3 and MLTC-1

To authenticate both cell lines we conducted STR profiling using the CellCheck™ Mouse 19 panel, which is designed to target 19 mouse-specific STR loci. The analysis revealed that both cell lines had the expected characteristic profile that is unique to them (**Supplementary Table 4**). Specifically, the cell line MLTC-1 showed 100% identity to the profile deposited in the Cellosaurus database. The TM3 cell line showed >80% similarity to previous STR profiles reported for this cell line, meeting the standard threshold to consider two profiles as identical.

### 3.3 Lipid droplets in Leydig cells

**Figure 4:**
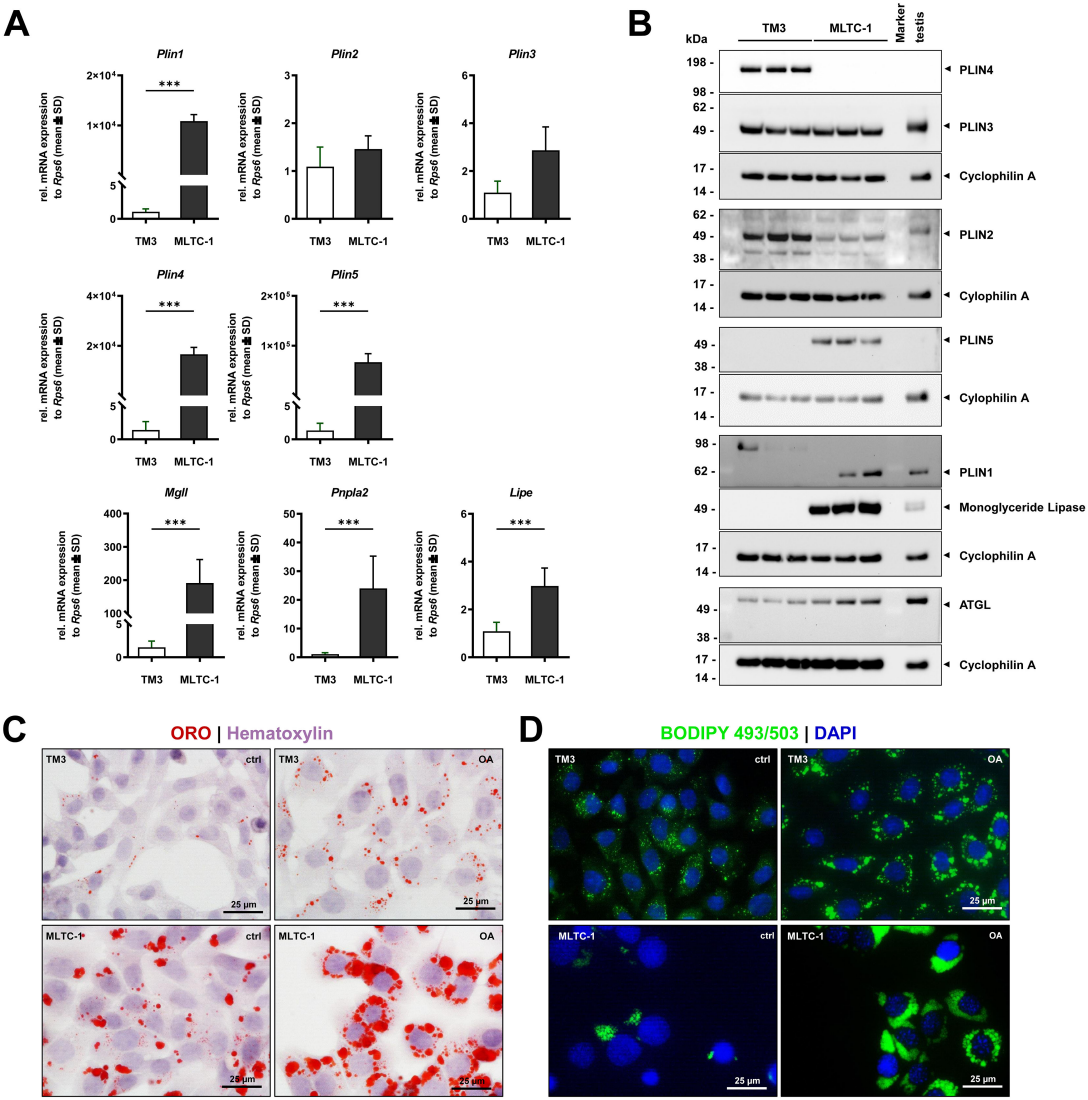
Markers associated with LDs in TM-3 and MLTC-1 LCs. **(A)** Basal mRNA expression of various LD-associated markers (*Plin1*, *Plin2*, *Plin3*, *Plin4*, *Plin5*) and lipolysis genes (*Mgll*, *Pnpla2*, *Lipe*) was compared between TM-3 and MLTC-1 cells using RT-qPCR. Relative mRNA expression was normalized to *Rps6* expression, and values are presented relative to TM3 cells. Data (from n=3 independent experiments) are shown as mean ± SD. Statistical analysis was performed using a Student’s *t*-test. Significant differences between the cell lines are denoted with asterisks: ***p < 0.001. **(B)** Expression of LD-associated and lipolytic proteins was compared between MLTC-1 and TM3 cells through Western blot analysis. Cyclophilin A expression was used as an internal loading control for protein analysis. TM3 and MLTC-1 cells were either stimulated with 0.25 mM oleic acid (OA) for 24 hours or left unstimulated before LD staining with Oil Red O (ORO) **(C)** or with the fluorescence probe BODIPY 493/503 **(D)**. LD staining was combined with nuclear counterstain using either haematoxylin or DAPI. Images in C and D were captured at 600× magnification, and scale bars represent 25 µm.

The presence of intracellular LDs is a characteristic feature of steroid-producing LCs (38). These organelles serve as storage for cholesterol in the form of cholesteryl esters that are required for steroid biosynthesis (38). Distinct proteins are involved in the formation, stabilization and cellular utilization of LDs, which change under different physiological conditions (38–40). Both cell lines express various genes associated with LD formation, transport and processing (**Supplementary Table S1**). One important family of molecules associated with LDs is the PAT (Perilipin/Adipophilin/TIP47) family, also known as the Perilipin (PLIN) family (38–41). Transcriptomic analysis revealed similar expression of *Plin2* and *Plin3* in TM3 and MLTC-1 cells. In contrast, *Plin1*, *Plin4* and *Plin5* expression was only found in MLTC-1 cells. Both cell lines express acetyl-Coenzyme A acetyltransferase 1 (*Acat1*), which is important for counteracting the toxicity of high free cholesterol levels by esterifying it (38). Genes for lipolysis were also detected in both cell lines, but patatin-like phospholipase domain containing 2 (*Pnpla2*), hormone sensitive lipase (*Lipe*), and monoglyceride lipase (*Mgll*) were found in higher rates in MLTC-1 cells. Real-time quantitative PCR (RT-qPCR) data confirmed the differences observed in the RNA-seq dataset (**Figure 4A**). Protein analysis by Western blot validated strong levels of PLIN2 and PLIN3 in both cell lines, whereas PLIN5 protein was solely expressed in tumorigenic MLTC-1 LCs (**Figure 4B**). In addition, levels of monoglyceride lipase were below the detection level in TM3 (**Figure 4B**). MLTC-1 cells express significantly higher levels of adipocyte triglyceride lipase (ATGL) mRNA (encoded by the *Pnpla2* gene) compared with TM3 cells, but only slightly more ATGL protein (**Figures 4A, B**).

It is noteworthy that commonly used housekeeping proteins for Western blot analysis such as β-actin and glyceraldehyde-3-phosphate dehydrogenase (GAPDH) differ not only at the mRNA level (see **Supplementary Table 3**) but also significantly at the protein level. Several frequently used proteins loading controls are shown in **Supplementary Figure 2**. Protein amounts were equally loaded and visualized on the membranes using Ponceau S staining. However, while β-actin, α-tubulin, and GAPDH are expressed at different levels in TM3 and MLTC-1, heat shock protein 90 (HSP90), Cyclophilin A, and Connexin 43 show similar levels in both cell lines and are therefore more suitable as loading controls in Western blotting in this context.

As depicted in **Figure 1**, light-microscopic refractive inclusions can be observed in the cytoplasm of both cell lines, most likely representing LDs. TEM confirmed the presence of LDs. ORO staining revealed LDS in the cytoplasm of both cell lines under standard conditions, with MLTC-1 displaying higher amount of LDs than the TM3 cells (**Figure 4C**). Stimulation with 0.25 µM OA for 24 hours showed an increase in lipid production or storage in both cell lines, validated by BODIPY 493/503 staining (**Figure 4D**). Additionally, after OA treatment, a strong perinuclear and cytoplasmic localization of LDs was observed in both cell lines (**Figure 4C, D**). TEM allowed for a closer observation of LD structure and formation, showing enhance presence of LD in both TM3 (**Supplementary Figures 3A–C**) and MLTC-1 cells (**Supplementary Figures 2D, E**) when stimulated with OA. Furthermore, organelles in the cells interacted with each other, forming associations between mitochondria, ER, and LDs (**Supplementary Figure 3**). The presence of elongated and larger mitochondria in TM3 cells suggests the possibility of mitochondrial fusion (**Supplementary Figure 3C**). In summary, using different methods, it was confirmed that both cell lines contain LDs in their cytoplasm and express different LD-associated markers, characteristic of LCs.

### 3.4 Differences in steroidogenic enzymes in Leydig cells

Studies have shown that steroid hormone synthesis occurs on LDs, with the movement of these organelles playing a role in reactions in LCs (40). There are conflicting reports in the literature regarding the expression of steroidogenic enzymes in TM3, which are necessary for proper hormonal production (10, 11, 16, 17). The transcriptomic dataset obtained in this study, along with the verification and confirmation of the findings through RT-PCR, RT-qPCR at the mRNA level, and Western blots at the protein level will serve as the basis for further studies with these cells.

**Figure 5:**
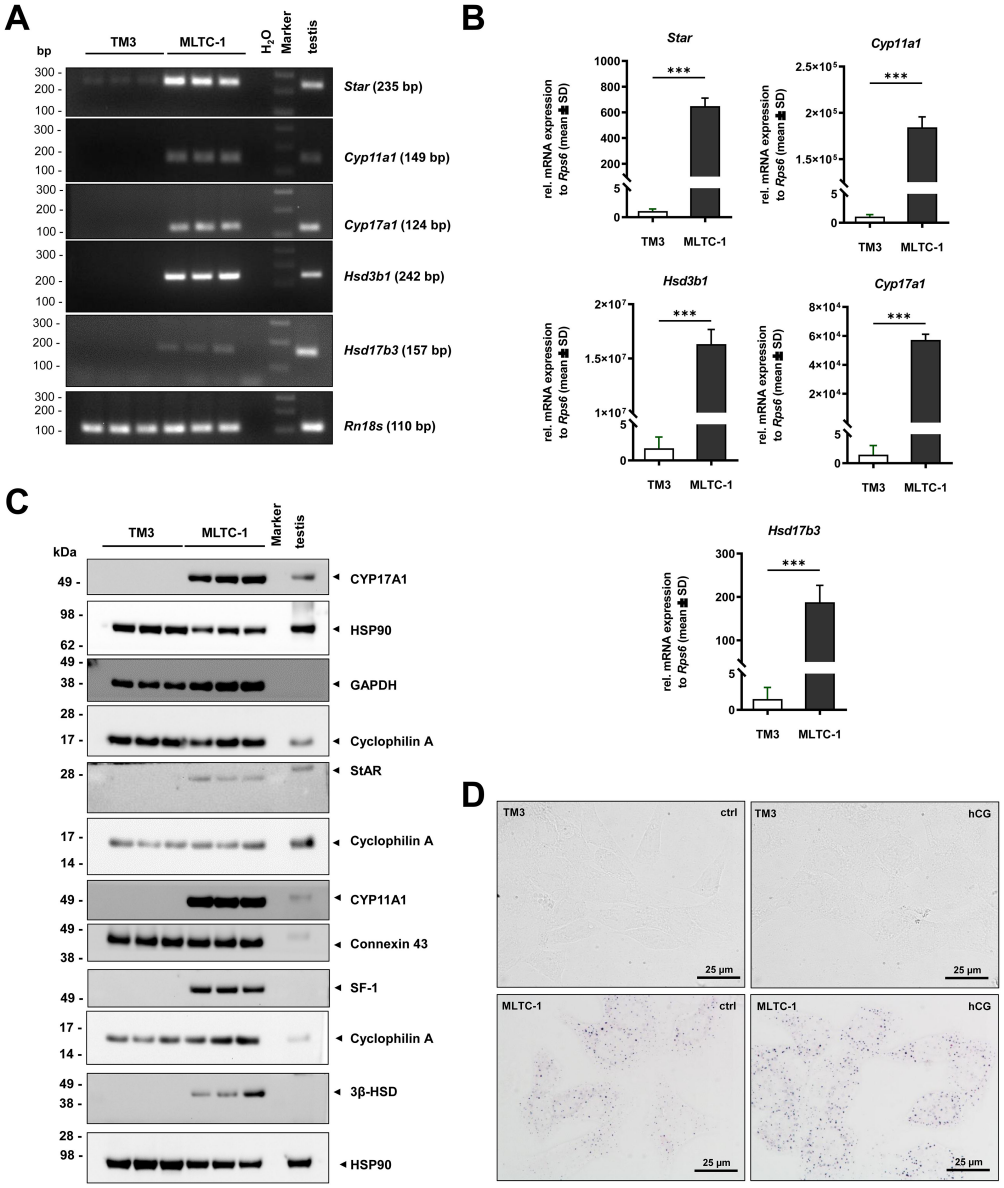
Marker of steroidogenesis in TM-3 and MLTC-1 LCs. **(A)** Basal mRNA expression of key enzymes of steroidogenesis was compared between TM-3 and MLTC-1 cells by RT-PCR with testis tissue serving as a control. **(B)** Data was confirmed by RT-qPCR. Respective genes are shown as fold change normalized to *Rps6*. The data are presented as mean ± SD. The statistical analysis used either an unpaired Student’s *t*-test or a Mann-Whitney test. Significant differences between groups are indicated by asterisks, ***p < 0.001. **(C)** Protein expression of selected steroidogenic markers was detected in TM3 and MLTC-1 cells with testis tissue as a control by Western blot analysis. All membranes were probed with antibodies to detect HSP90, GAPDH, Connexin 43 or Cyclophilin A to ensure equal protein loading. **(D)** 3β-HSD activity was visualized in TM3 and MLTC-1 cells using an established histological staining procedure. Before staining, cells were either treated with 5 IU/ml human chorionic gonadotropin (hCG) or left untreated for 24 hours. Blue precipitates within the cytoplasm of MLTC-1, but not in TM3 cells, indicate a positive reaction with and without hCG treatment. Images were captured at 600× magnification and scale bars equal 25 µm.

The RNA-seq data provide a valuable initial overview of the expression of steroidogenesis enzymes (**Supplementary Table 3**). To confirm this data, specific primers targeting important steps of steroidogenesis were used in RT-PCR and RT-qPCR. Our data show that MLTC-1 cells have significantly higher levels of key steroidogenic enzymes (*Star, Cyp11a1, Cyp17a1, Hsd3b1, Hsd17b3*) compared to TM3 cells (**Figures 5A, B**). Other enzymes associated with steroidogenesis such as cytochrome P450, family 19, subfamily a, polypeptide 1 (*Cyp19a1)*, which catalyzes the synthesis from androgens to estrogens, were also analyzed (**Supplementary Figure 4**), showing significantly lower levels in TM3 cells. Although TM3 cells contain trace amounts of *Star*, restricting steroid production, all other enzymes in these cells remain undetectable at the mRNA and protein levels (**Figures 5A–C**). Furthermore, we examined the expression of insulin-like factor 3 (*Insl3*) in both cell lines. *Insl3* is not only crucial for testicular descent, but also serves as a marker for the function and activity of LCs. Interestingly, we found no detectable transcript of *Insl3* in TM3 cells, while MLTC-1 cells showed over 100,000 TPM (**Supplementary Table 3**). This finding was confirmed through RT-PCR and RT-qPCR, demonstrating a significantly higher expression of *Insl3* in MLTC-1 cells (**Supplementary Figure 4**). Additionally, steroidogenic factor 1 (SF-1), encoded by nuclear receptor subfamily 5, group A, member 1 (*Nr5a1*), a major regulator of steroidogenic enzymes, is strongly expressed in MLTC-1 cells but absent in TM3 cells (**Figure 5C**). Western blot analyses not only confirm the presence of transcripts in MLTC-1 but also the successful translation of steroidogenic enzymes such as StAR, CYP11A1, CYP17A1 and 3β-HSD (**Figure 5C**). Despite low levels of transcripts for *Hsd3b1* in TM3 cells, there was no protein expression of 3β-HSD in Western blot analysis.

**Figure 6:**
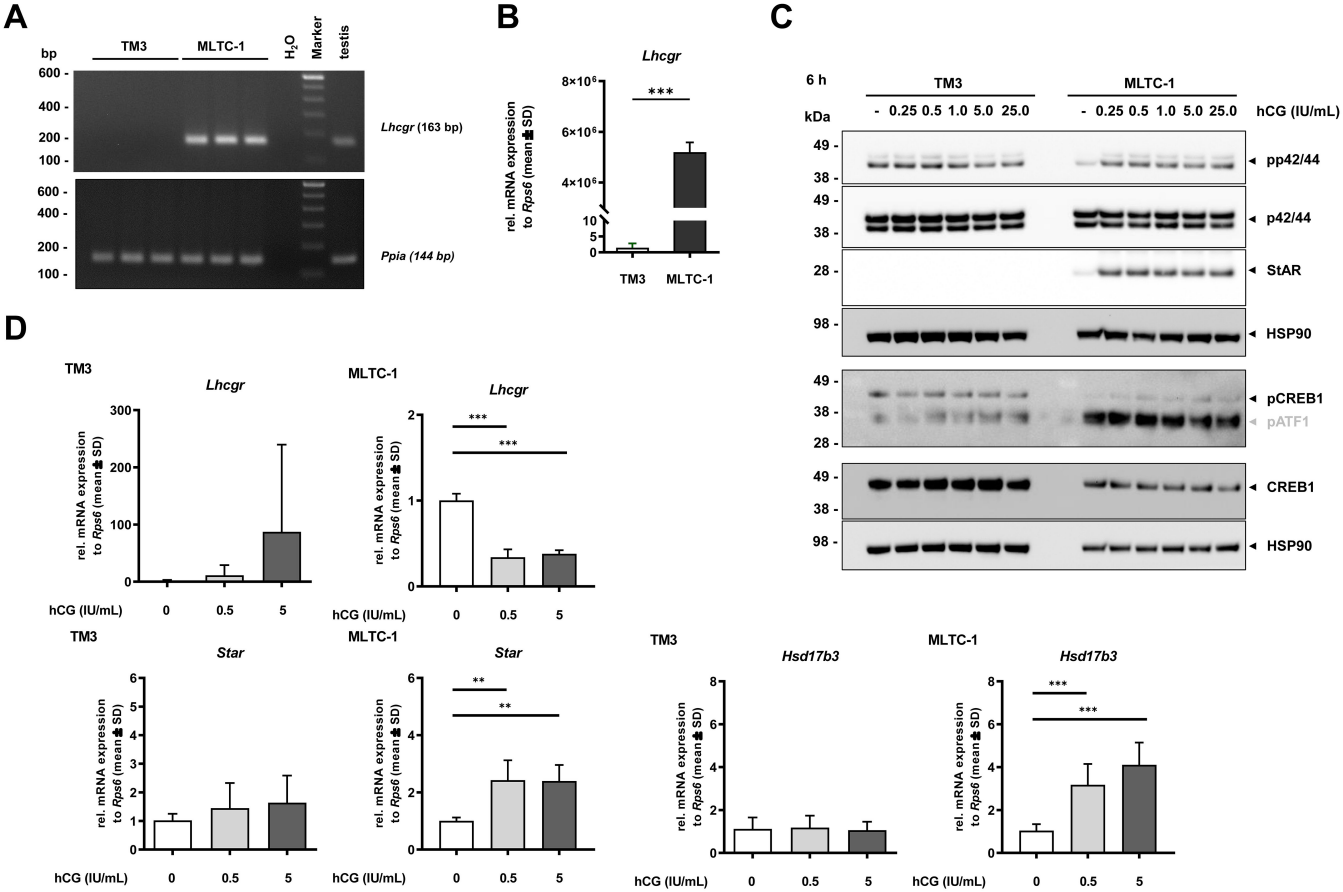
Induction of steroidogenesis by hCG stimulation in TM-3 and MLTC-1 LCs. **(A)** Basal mRNA expression of *Lhcgr* was studied in TM-3 and MLTC-1 cells by RT-PCR with testis tissue as a control. **(B)** Data was confirmed by RT-qPCR. *Lhcgr* expression was normalized to *Rps6* and data are displayed in relation to TM3 cells. Student’s *t*-test was used for statistical analysis. There was a significant difference in *Lhcgr* expression between TM3 and MLTC-1 cells, indicated by asterisks, ***p < 0.001. **(C)** Both cell lines were treated with or without hCG (0.5 IU/mL or 5 IU/mL) for 6 hours and RT-qPCR was used to detect changes in *Lhcgr, Star* or *Hsd17b3* expression. Relative mRNA expressions are normalized to *Rps6* and displayed in relation to untreated cells. The data in C and D are presented as mean ± SD. For statistical analysis in C, one-way ANOVA was used. **(D)** TM3 and MLTC-1 cells were treated with different concentrations of hCG (0, 0.25, 0.5, 1.0, 5.0 or 25 IU/mL) for 6 hours, and protein expression of different markers (Phospho-p42/44, total p42/44, StAR, phospho or CREB1) was detected by Western blot analysis. Please note that the phospho-CREB1 antibody detects phosphor-ATF1 as well (illustrated in grey letters). HSP90 expression was used to ensure equal protein loading. MLTC-1 cells but not TM3 cells show induction of steroidogenic markers upon hCG treatment. **p < 0.01.

In histological staining for 3β-HSD activity, a strong signal (blue precipitates) was found in the cytoplasm of MLTC-1 cells. However, no 3β-HSD activity was detected in TM3 cells. It is well known that steroidogenesis can be induced by hCG, which binds to the luteinizing hormone receptor (encoded by *Lhcgr*) (12). To investigate whether steroidogenesis can be induced in TM3 cells, the cells were treated with 5 IU/mL hCG for 6 hours, and then the activity of 3β-HSD was examined through staining. Our data clearly show that, even after hCG treatment, no 3β-HSD activity is detectable in TM3 cells (**Figure 5D**). In contrast, enhanced positive staining was observed in MLTC-1 cells when treated with hCG for 6 hours. This was unexpected, as Mather et al. reported in 1982 that TM3 cells are responsive to hCG treatment and positive for LHCGR expression. However, our RNA-seq data showed no transcripts for *Lhcgr* in TM3 cells. This finding was verified though RT-PCR and RT-qPCR analysis (**Figures 6A, B**). A concentration series (ranging from 0.25 to 25 IU/mL hCG) after a 6-hour stimulation period, confirmed activation of phospho-ERK1/2 (p42/44) and phospho-CREB1 in MLTC-1 cells, in addition to StAR, but not in TM3 cells (**Figure 6C**). No dose-dependent increase in the expression of any of the examined markers was observed in this series, with expression in TM3 cells remaining unchanged.

Treatment with hCG for 6 hours did not induce any change in TM3 cells at the mRNA level, but there was a significant increase in *Star* and *Hsd3b17* in MLTC-1 cells (**Figure 6D**). Conversely, a substantial reduction in *Lhcgr* indicated a clear induction of the LHCGR pathway in MLTC-1 cells, but not in TM3 cells. This effect persisted, with *Lhcgr* still significantly reduced in the cells even after 24 hours of treatment with hCG (**Supplementary Figure 5A**). *Hsd17b3* levels were significantly reduced after 24 hours for both concentrations of hCG. The expression of *Star* mRNA showed no change after 24 hours, while the amount of StAR protein remained markedly increased (**Supplementary Figures 5A, B**).

It is known that hCG has a significant impact on the organization and structure of organelles, including mitochondria and the ER (42, 43). We were able to demonstrate the activation of steroidogenesis in MLTC-1 cells and observe morphological changes after treatment with hCG. Light microscopy showed more elongated, stretched MLTC-1 cells after 24 hours of stimulation with hCG (**Supplementary Figure 5C**). In contrast, TM3 cells maintained their polygonal shape after hCG treatment. Additionally, F-actin stress fibers, stained with Phalloidin-Rhodamine confirmed these observations (**Supplementary Figure 5D**).

**Figure 7:**
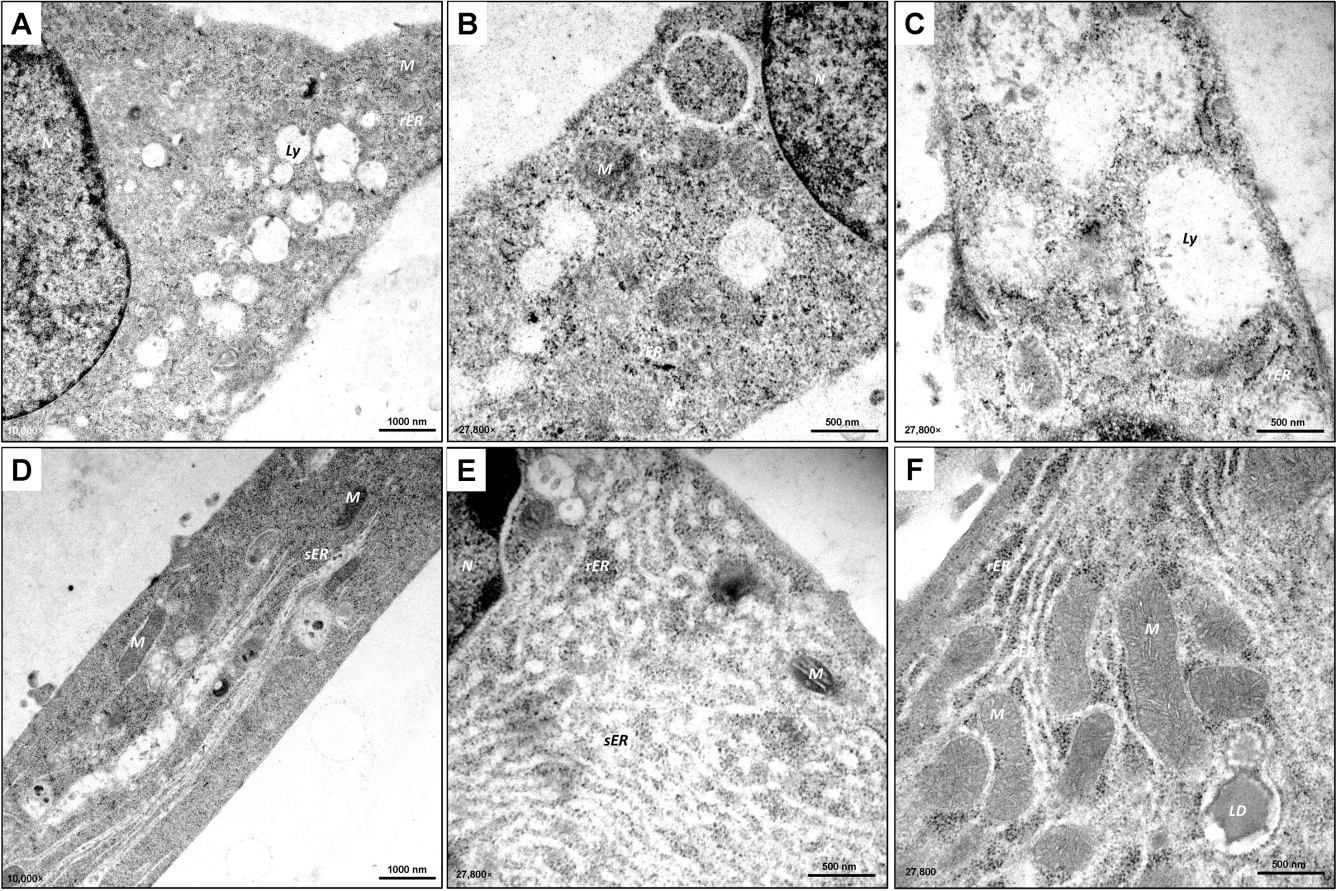
Ultrastructural analysis of TM3 and MLTC-1 cells stimulated with hCG. **(A–F)** Following a 24-hour treatment with hCG, TEM was performed on TM3 **(A–C)** and MLTC-1 cells **(D–E)**. The ultrastructural analysis revealed the presence of lytic vesicles (*Ly*) in TM3 cells. In hCG-treated MLTC-1 cells, a significant amount of smooth endoplasmic reticulum (*sER*) was observed in the form of bundles or whorls consisting of ribosomal-free membranes with expanded lumens (white). Mitochondria (*M*) were found in in close proximity to both the sER and rough endoplasmic reticulum (*rER*). Abbreviations: nucleus (*N*). TEM images were taken at 10,000× **(A, C)** and 27,800× **(B, C, E, F)** with scale bars representing 1000 nm or 500 nm.

Interestingly, TEM analysis revealed differences in the altered structure of cellular organelles in both cell lines (**Figure 7**) depending on hCG-responsiveness. mRNA and protein analysis showed that TM3 cells did not respond to hCG stimulation with an increase in steroidogenesis (**Figure 6**). However, at the ultrastructural level, a significant increase in lytic vesicles was observed (**Figures 7A–C**). In contrast, MLTC-1 cells exhibited rearrangements of cellular organelles (**Figures 7C–E**). The cytoplasm of hCG-treated MLTC-1 cells showed permeation by smooth endoplasmic reticulum (sER), appearing as bundles, tubes, or whorls of ribosome-free membranes with empty lumens. Furthermore, the rER and sER were present in close proximity around the mitochondria after 24 hours of hCG treatment, indicating interaction between the organelles in the cells. Overall, all these structural changes in MTLC-1 cells following hCG-treatment align with data from RT-qPCR or Western blot analysis, confirming a strong activation of steroidogenesis in these cells. This data demonstrates that MLTC-1 cells, compared to TM3 cells, exhibit a functional response to hormonal stimuli, a characteristic of LCs that is essential for *in vitro* systems.

## 4 Discussion

There is an urgent need for tools to identify substances that interfere with steroidogenesis potentially leading to male infertility as male reproductive health has significantly declined in recent decades (44). In terms of development, it is known that there are at least two distinct generations of LCs. Early in prenatal development, fetal LCs play a key role in androgen production, responsible for masculinization during fetal and neonatal life. The number of fetal LCs decreases after birth and they are later replaced by adult LCs, which first appear around postnatal day 10 and increase in number until puberty (45–48). The increase in the number of LCs is controlled by the steroid hormones LH and follicle stimulating hormone (FSH) (45). Adult LCs produce testosterone, which impacts spermatogenesis, development of secondary sexual characteristics, and muscle and bone health (49). Despite their high medical relevance, access to primary human LCs remains severely limited due to limited tissue sources and their rapid dedifferentiation *in vitro* (2, 50). Purified primary LCs from rodents could serve as a suitable cell-based model to study steroidogenesis, but preparations are costly, yield is often low, and trained personnel are needed (50). Immortalized cell lines serve as a tool to study mechanisms and signaling pathways of steroidogenesis which can be genetically manipulated more easily (10–12). However, these cell lines have limitations as they are often isolated from tumors or have steroidogenic pathways that are not completely activated compared to primary cells (12, 50). Additionally, only well-characterized cell-based models can be used for reliable and reproducible approaches. Therefore, comprehensive cell authentication is a mandatory step not only in endocrine research (22).

Although TM3 and MLTC-1 are described as LC lines, they have completely different origins. TM3 cells were derived from the testes of pre-reproductive aged mice (11 to 13 days old) (16, 17), while MLTC-1 cells stem from a transplantable LC tumor in mice (M548OP) (18). There is currently debate as to whether LC lines such as TM3 or MLTC-1 are suitable model systems for primary LCs, as all permanent cells do not express all enzymes of testosterone biosynthesis in sufficient levels *in vitro* (10–13). To facilitate a robust and comprehensive analysis, a comparative morphological study, including light and electron microscopy, was conducted between the two cell lines, followed by a thorough transcriptional analysis.

*In vivo*, LCs, are found in clusters within the interstitium of the testis, and are characterized by the presence of relevant hormonal receptors and steroidogenic enzymes. They possess a morphology typical of steroid-producing cells including abundant smooth endoplasmic reticulum, mitochondria with tubular cristae, and are rich in lipid droplets, which serve as the source of cholesterol for androgen production (1). TM3 cells, in line with their original description, exhibit an adherent growth behavior in monolayers until confluence with an epithelial morphology (16). In comparison, MLTC-1 cells form islands or clusters with an epithelial morphology but these monolayers do not become fully confluent, as reported by Rebois in 1982 (18). Microscopic analysis of both cell lines revealed that TM3 and MLTC-1 cells exhibit polygonal LC characteristics (46) with tight cell-cell contacts, visible in the F-actin structure by a Phalloidin probe. Transcriptomic analysis also revealed various markers for cellular contacts and communication in TM3 and MLTC-1 cells. Cell-cell contacts and communication are essential for the interplay between testicular cells in maintaining spermatogenesis (35). Mather et al. in 1982 originally pointed out that TM3 cells contain microvilli on the surface of the cells (17), which were also found in MLTC-1.

Crucial for cellular communication is the gap junction protein connexin 43 (encoded by *Gja1*) which is abundantly expressed in various testicular cells, including LCs (51, 52). The RNA-seq data obtained in the study reveals high levels of *Gja1* in both cell lines, consistent with previous reports highlighting the important role of connexin 43 in intracellular communication in TM3 cells (53). Interestingly, in contrast to the expression patterns of conventional housekeeping proteins such as GAPDH or β-actin, our study reveals that connexin 43 (as well as HSP90) exhibits equal expression levels in TM3 and MLTC-1 at the protein level. This observation suggests that connexin 43 is a promising candidate for protein analysis comparing these two cell lines.

The differentiation of adult LCs is evident in their morphology, which shows abundant sER, LDs and mitochondria, often with tubular cristae, common in steroidogenic cells (45, 54). Ultrastructural analysis of TM3 and MLTC-1 cells in this study demonstrates that both cell lines exhibit these typical LC-associated characteristics. The architecture of the mitochondria and its interaction with other organelles, such as the sER, is closely linked to its steroidogenic function in cholesterol synthesis and transport within the cell (45). Additionally, upon stimulation with hCG, massive rearrangement of cellular organelles was observed in MLTC-1 cells though TEM. The mitochondria, highly electron-dense ribosomes, and the sER are closely associated, as essential steps of steroidogenesis are maintained by enzymes on the mitochondrial membrane (3). In this context, it was shown that steroid synthesis in MA-10 cells, another tumorigenic immortal LC line, involves changes in mitochondrial fusion, controlled in a hormone-dependent manner, increasing ER-mitochondria communication (55, 56).

Activation of steroidogenesis leads to the formation of substantial bundles or whorls-like sERs formations with empty lumens extending through the cytoplasm of the cells. This restructuring indirectly provides insights into the activation of key components of steroidogenesis, such as StAR, facilitating cholesterol transport, and 3β-HSD involved in pregnenolone processing (3). Prolonged hormone treatment acts as a cellular stress factor, triggering ER stress to maintain proper folding capacity (43, 57–59). Consistent with our data, Park and colleagues reported a decline in steroidogenic enzymes like *Hsd17b3* after 24 hours of hCG treatment in MLTC-1 cells (43). Ultrastructural analysis of hCG-treated TM3 cells reveals numerous lytic vesicles, potentially indicative of lysosomes attempting to break down substances due to the absence of *Lhcgr*, preventing normal steroidogenic processes.

The presence of intracellular LDs is a characteristic feature of steroid-producing cells (38). In LCs, these organelles primarily serve as storage for cholesterol in form of cholesteryl esters that are required for steroid biosynthesis (38, 39). Herein, TEM and staining provide clear evidence that both LC lines contain endogenous LDs in their cytoplasm. In particular, the MLTC-1 line has a significantly larger amount of LDs than the TM3 line. As a positive control OA was used as it is known that fatty acids induce lipid accumulation in cells (60). Treatment with OA resulted in a dramatic increase in LDs in both cell lines.

Ten years ago, Yamaguchi and colleagues characterized the lipid droplet profile of MLTC-1 and suggested that LDs are actively involved in steroidogenesis providing sites for enzymatic activity, as perinuclear LD distribution dispersed upon LH treatment (40). It was reported that the MLTC-1 line exhibits several different LD-associated proteins including lipase modulators, enzymes for lipolysis, proteins for vesicular trafficking, chaperones and many others (40). These data are in line with our transcriptomic analysis, RT-qPCRs and Western blot analysis. In TM3 cells, LDs have not been comprehensively characterized. Our data provides evidence that both cell lines contain high levels of *Lipe*, which is known as HSL and hydrolyzes free cholesterol from cholesteryl esters, providing a substrate for steroidogenesis (38). Further enzymes for lipolysis (*Pnpl2*, *Mgll*) are present in both cell lines, but at lower levels in TM3 cells. In contrast, TM3 and MLTC-1 cells possess transcripts for *Acat1*, an important enzyme that regulates the availability of free cholesterol by esterifying it to counteract the toxicity of high cholesterol levels (38). MLTC-1 and TM3 cells exhibit high mRNA and protein levels of PLIN2 and PLIN3, two important LD-associated proteins required for stabilization and LD transport (41, 61). However, in TM3 cells, there were no transcripts of *Plin1* or protein present, which is an important coat surface protein (38). Interestingly, *Plin5* mRNA and protein were solely detectable in MLTC-1 cells. In recent years, PLIN5 has gained attention as it recruits mitochondria to the LD surface, inducing physical contact between these organelles and regulating the hydrolysis of LDs (62). These findings are consistent with the reduced steroidogenic capacity and low levels of key enzymes in TM3 cells.

However, our data provide clear evidence that the currently available TM3 cells are not suitable to study *Lhcgr*-mediated signaling pathways or downstream steroidogenic processes, as the cells lack *Lhcgr* expression. The *Lhcgr* is one of the most important receptors in the stimulation, differentiation and maturation of LCs *in vivo* as it mediates luteinizing hormone (LH) signaling from the pituitary to the testes (63). In humans, LHCGR can potentially be activated by hCG, which is a well-known agonist with crucial physiological functions, particularly during gestation (63, 64). Different inactivating mutations in the G protein-coupled LHCGR are known to result in insufficient LC differentiation, leading to LC hypoplasia, a disorder that results in the feminization of males (65, 66). Additionally, TM3 cells show vanishingly small or no levels of important regulatory proteins and transporters such as *Star*, *Cyp11a1*, *Cyp17a1*, *Hsd3b1* or *Hsd17b3*. Furthermore, the cells showed no response to hCG stimulation, neither on a phenotypical level nor on a molecular level. This contrasts with the original description of the cells by Mather and colleagues (16, 17). However, our results are in line with data from others who found low to no *Cyp17a1* or *Hsd17b3* expression in TM3 cells (10, 13). As early as 2007, it was questioned whether TM3 cells are truly LCs, as they were isolated from a mixed culture of testicular cells and there is no comprehensive transcriptomic or proteomic analysis in this regard (12).

One of the first and still most important characteristics of LCs that distinguishes them from other testicular cells is the histochemical detection of 3β-HSD, which was first discovered in 1958 by Wattenberg (28). A study in rabbit testis provided the initial evidence that interstitial cells are the primarily source of androgen production in vertebrates (28). A modified staining procedure was utilized to evaluate 3β-HSD activity (5, 6) in murine TM3 and MLTC-1 cells. The results showed moderate 3β-HSD activity in MLTC-1 cells, but no staining in TM3 cells, which was consistent with the presented transcriptomic and protein analysis data. Another crucial marker for the activity and differentiation of LCs is INSL3, as depletion causes cryptorchidism in mice (67, 68). INSL3 expression has been found in fetal and adult LCs, being expressed in most mammals, including humans (69, 70). However, our data demonstrate strong *Insl3* expression only in MLTC-1 cells, but not in TM3 cells.

Due to the extended cultivation period, it is entirely possible that the cell lines have changed since their development. However, genetic changes due to high passage numbers or cross-contamination during the period in which the cell lines were developed are unfortunately still common today (71, 72). A decline in LHCGR expression has been reported for other cell lines upon prolonged cultivation or inappropriate culturing conditions (12, 15). Hirakawa and coworkers noticed a massive reduction in the density of LHCGR expression and a barely noticeable response to hCG in MA-10 cells (15). MA-10 cells are another important and widely studied model for immortalized LC lines. These cells are closely related to MLTC-1 cells, but were isolated independently from the M5480 tumor in 1981 by Ascoli (14). The authors found that adjusting the culture conditions diminished this effect. They also demonstrated through genetic manipulation of the cells that transient overexpression of LHCGR is functionally important (15). However, our RNA-seq data demonstrate that MLTC-1 still possesses more than 50,000 TPM of *Lhcgr*. In contrast to TM3 cells, MLTC-1 expresses high levels of the androgen receptor (encoded by *Ar*) and even transcripts for *Hsd17b3* which encodes the enzyme 17β-HSD3 that mediates the final step of steroidogenesis. In the early stages of testis development, up to 20 days, Sertoli cells in the seminiferous tubules express 17β-HSD3, which is later restricted to the interstitial LCs (73). It is well known that all available LC lines, such as MLTC-1 and MA-10 cells, exhibit impaired steroidogenesis potentially due to the immortalization process (10–12, 14, 15). Consequently, the activation of the LHCGR signaling pathway can only be studied up to the progesterone step. Nevertheless, studies show that a small but detectable amount of testosterone could be found in MLTC-1 by radioimmunoassay after hCG stimulation (20).

When the data from the entire study is compiled, it becomes clear that TM3 cells barely express any markers for LCs and steroidogenesis under these culture conditions. It should be noted, as already mentioned, that the line was obtained from testicular cells of 11-13-day-old mice, which are more similar to a pre-pubertal type (16). When generating a 3D-based model for testicular toxicity studies using TM3 cells, Sychrová and coworkers compared immature and mature LC markers in these cells. They found strong expression for immature markers *Arl13b* (ADP-ribosylation factor-like 13B), *Dlk1* (delta like non-canonical Notch ligand 1) and *Wt1* (WT1 transcription factor) (13). Our RNA-seq analysis revealed transcripts for *Arl13b* and *Wt1* in TM3, but no *Dlk1*. Furthermore, *Arl13b* and *Dlk1* were present in adult MLTC-1 cells. Whereas the authors found strong SF-1 mRNA, encoded by *Nr5a1* in TM3 cells, our transcriptomic and Western blot analysis revealed no detectable levels of SF-1 in TM3 cells. These differences show that, despite purchasing from the same company in recent years and under comparable cultivation conditions, the results can vary and potentially the expression of genes alters during prolonged cultivation.

As highlighted by Engeli et al., there is an urgent need for mouse and, even more importantly, human LC lines expressing the full complement of enzymes necessary for steroidogenesis in order to conduct pharmacological and toxicological screening (10). However, it is crucial that the cell lines used in publications are screened for marker expression and that their purity is verified using STR (74), which is well established for cell lines such as MLTC-1 and TM3.

## 5 Conclusion

Although the tumorigenic MLTC-1 LC line has some limitations in representing a model with a complete set of steroidogenic enzymes, these cells possess several pivotal characteristics of adult mouse LCs on a morphological and molecular level. The presence of the *Lhcgr* qualifies these cells for investigation of the activation of steroidogenesis (upstream) and downstream signaling pathways. Additionally, the MLTC-1 cell line is suitable for investigating structural changes in endocrine-active substances, a critical consideration in the context of declining male reproductive health. Given the established correlation between cholesterol metabolism and steroidogenesis, further research is warranted on LDs, LD-associated proteins, and their precise composition within cells. Through STR profiling, we confirmed the identity and NGS has elucidated the gene expression profile of both cell lines comprehensively. Although this study focused on one of the two most commonly used tumorigenic LC lines and did not examine MA-10 cells, it can be assumed that they respond equally to hCG treatment as MLTC-1 does, given their similar origin and optimized cultivation conditions (15). A comprehensive comparison of these two cell lines, as well as an investigation of other species such as rat LCs (e.g. R2C cells), is essential for endocrine research. Working with these cell lines requires a comprehensive molecular and genetic characterization including STR profiling. Even though our data clearly show that TM3 cells are rather unsuitable for studying steroidogenesis as they lack expression of specific enzymes and the crucial LC marker *Insl3*, these cells exhibit typical morphological characteristics of LCs, including LD and key LD-associated proteins. The potential for genetic manipulation of *Lhcgr* or key steroidogenic enzymes could serve as a tool for optimizing these cells. Moreover, recent studies demonstrate that cultivation as a 3D spheroid is physiologically closer to *in vivo* conditions, highlighted by alterations in steroidogenic markers in TM3 cells (13). It is evident that this has considerable potential in the field of endocrine research for other cell lines, such as MLTC-1 cells.

## Data Availability

The original contributions presented in the study are included in the article/**Supplementary Material**, further inquiries can be directed to the corresponding author/s.

## Supplementary material

Supplementary Table 1 Primary and secondary antibodies used for Western blot analysis

Supplementary Table 2 Primers used in this study.

Supplementary Table 3 Gene expression in MLTC-1 und TM3 cells.

Supplementary Table 4 Short tandem repeat (STR) profiling of TM3 and MLTC-1 cells.

Supplementary Figure 1 Mycoplasma testing.

Supplementary Figure 2 Loading controls for Western blot analysis in TM3 and MLTC-1 cells.

Supplementary Figure 3 Ultrastructural analysis of LC lines stimulated with oleic acid (OA).

Supplementary Figure 4 Additional markers of steroidogenesis in TM-3 and MLTC-1 Leydig cells.

Supplementary Figure 5 Effects of chorionic gonadotropin (hCG) stimulation in TM-3 and MLTC-1 Leydig cells.

Supplementary Figure 1 Mycoplasma testing. MLTC-1 and TM3 cell lines were tested for mycoplasma infection using a positive (+) control and a negative control medium (–). The internal control showed a 191 bp fragment with each probe, indicating that the PCR reactions were working correctly. Amplification of a 265–278 bp fragment, as seen in the positive control sample, confirmed the presence of a mycoplasma contaminant. Both cell lines were tested negative for *Mycoplasma* spp.

Supplementary Figure 2 Loading controls for Western blot analysis in TM3 and MLTC-1 cells. Endogenous basal protein was harvested from TM3 and MLTC-1 cells cultured for 3 days, purified, quantified and subjected to Western blot analysis with testis tissue as a control. Equal amounts of protein (40 µg) were visualized on the membranes using Ponceau S staining. β-actin, α-tubulin and glycerinaldehyd-3-phosphat-dehydrogenase (GAPDH) showed differences in expression between the cell lines. The expressions of heat shock protein 90 (HSP90), Connexin 43 and Cyclophilin A appear to be more suitable as loading controls when comparing protein expression levels in TM3 and MLTC-1. The lanes represent samples from n= 3 independent experiments per cell line.

Supplementary Figure 3 Ultrastructural analysis of LC lines stimulated with oleic acid (OA). After stimulation with 0.25 mM OA for 24 hours, TM3 **(A-C)** and MLTC-1 **(D-E)** cells were subjected to transmission electron microscopy (TEM). The ultrastructural analysis shows an increase in lipid droplets (*LD*) in the cytoplasm of both cell lines, with the droplets being larger in MLTC-1 cells. Upon OA treatment, there was a close interaction between mitochondria (M) and endoplasmic reticulum (*ER*). TM3 cells displayed several lytic vesicles (*Ly*). Abbreviations used: nucleus (*N*), glycogen (*G*). TEM images were captured at 10,000× **(A, D)** and 27,800× **(B, C, E, F)** with scale bars representing 1000 nm or 500 nm.

Supplementary Figure 4 Additional markers of steroidogenesis in TM-3 and MLTC-1 Leydig cells. **(A)** Basal mRNA expression of several enzymes involved in steroidogenesis and LC marker *Insl3* were compared between TM-3 and MLTC-1 cells suing RT-PCR with testis tissue serving as a control. **(B)** The data was further validated using RT-qPCR and presented as fold change normalized to *Rps6*. The data is shown as mean ± SD. Statistical analysis was performed using an unpaired Student’s *t*-test or Mann-Whitney test. Significant differences between the cell lines are denoted by asterisks, *** *p* < 0.001.

Supplementary Figure 5 Effects of chorionic gonadotropin (hCG) stimulation in TM-3 and MLTC-1 Leydig cells. Both cell lines were treated with or without hCG (0.5 IU/mL or 5 IU/mL) for 24 hours and RT-qPCR was used to detect changes in *Lhcgr, Star*, or *Hsd17b3* expression. Relative mRNA expressions were normalized to *Rps6* and displayed in relation to untreated cells. The data are presented as mean ± SD. For statistical analysis in **(A)**, one-way ANOVA was used. **(B)** TM3 and MLTC-1 cells were treated with different concentrations of hCG (0, 0.25, 0.5, 1.0, 5.0 or 25 IU/mL) for 24 hours, and protein expression of different markers (Phospho-p42/44, total p42/44 and StAR) was detected by Western blot analysis. HSP90 expression was used to ensure equal protein loading. MLTC-1 cells, but not TM3 cells, showed induction of steroidogenic markers upon hCG treatment. **(C)** Light microscopy images of TM3 and MLTC-1 cells treated with 0, 0.5 or 5.0 IU/mL hCG for 24 hours were captured at 200× magnification, with a scale bar equaling 50 µm. **(D)** The F-actin cytoskeleton of Leydig cell lines treated with 5 IU/mL hCG for 24 hours was labeled with Rhodamine-Phalloidin (red), and nuclear counterstaining was done with DAPI (blue). Images were captured at 600× magnification with scale bars equaling 25 µm.
