# Supplementary material for: Molecular characterization of the murine Leydig cell lines TM3 and MLTC-1
Source: Front Endocrinol (Lausanne). 2025 Dec 16;16:1715307. doi: 10.3389/fendo.2025.1715307 (PMC12747838; doi:10.3389/fendo.2025.1715307)
Supplement: Supplementary Table 1 — Primary and secondary antibodies used for Western blot analysis [file Table1.docx]

| **Supplementary Table 1: Primary and secondary antibodies used for Western blot analysis** | | | | | | | |  |  |
| --- | --- | --- | --- | --- | --- | --- | --- | --- | --- |
| **Antibody** | **Catalog No.** | **RRID^1^** | **Dilution** | **Host, Clonality** | | **Company** | |  |  |
| 3β-HSD | sc-515120 | AB_2721058 | 1:500 | m, mAb | | Santa Cruz Biotech., CA, USA | |  |  |
| ATGL (30A4) | #2439 | AB_2167953 | 1:1,000 | r, mAb | | Cell Signaling Technology, Leiden, The Netherlands | |  |  |
| Collagen I | ab34710 | AB_731684 | 1:1,000 | r, pAb | | Abcam, Cambridge, UK | |  |  |
| Collagen III | 22734-1-AP | AB_2879158 | 1:1,000 | r, pAb | | Proteintech, Planegg-Martinsried, Germany | |  |  |
| Collagen IV | ab6586 | AB_305584 | 1:1,000 | r, pAb | | Abcam | |  |  |
| Connexin 43 (C-20)* | sc-6560-R | AB_638639 | 1:1,000 | r, pAb | | Santa Cruz Biotech. | |  |  |
| CREB (D76D11) | #4820 | AB_1903940 | 1:1,000 | r, mAb | | Cell Signaling Technology | |  |  |
| Cyclophilin A | #2175 | AB_2169116 | 1:1,000 | r, pAb | | Cell Signaling Technology | |  |  |
| CYP11A1 (D8F4F) | #14217 | AB_2631970 | 1:1,000 | r, mAb | | Cell Signaling Technology | |  |  |
| CYP17A1 (E6A7G) XP® | #94004 | AB_2800219 | 1:1,000 | r, mAb | | Cell Signaling Technology | |  |  |
| Fibronectin | AB1954 | AB_2105708 | 1:3,000 | r, pAb | | Sigma-Aldrich, Taufkirchen, Germany | |  |  |
| GAPDH (6C5) | sc-32233 | AB_627679 | 1:1,000 | m, mAb | | Santa Cruz Biotech. | |  |  |
| HSP90 (C45G5) | #4877 | AB_2233307 | 1:1,000 | r, mAb | | Cell Signaling | |  |  |
| Monoglyceride Lipase | sc-398942 | AB_3714782 | 1:500 | m, mAb | | Santa Cruz Biotech. | |  |  |
| p44/42 MAPK (Erk1/2) | #9102 | AB_330744 | 1:1,000 | r, pAb | | Cell Signaling Technology | |  |  |
| Perilipin (D1D8) XP | #9349 | AB_10829911 | 1:1,000 | r, mAb | | Cell Signaling | |  |  |
| Perilipin 4 | ABS526 | AB_3714783 | 1:1,000 | r, pAb | | Sigma-Aldrich | |  |  |
| Perilipin 5 | 26951-1-AP | AB_2880699 | 1:1,000 | r, pAb | | Proteintech | |  |  |
| Perilipin-2/ADFP | NB110-40877 | AB_787904 | 1:500 | r, pAb | | Novus Biologicals, Bio-Techne GmbH, Wiesbaden Nordenstadt, Germany | |  |  |
| Perilipin-3/TIP47 | NB110-40765 | AB_715112 | 1:1,000 | r, pAb | | Novus Biologicals | |  |  |
| Phospho-CREB1 (10E9) | sc-81486 | AB_1125727 | 1:500 | m, mAb | | Santa Cruz Biotech. | |  |  |
| Phospho-p44/42 MAPK (Erk1/2) (Thr202/Tyr204) | #9101 | AB_331646 | 1:1,000 | r, pAb | | Cell Signaling Technology | |  |  |
| StAR (D10H12) XP® | #8449 | AB_10889737 | 1:1,000 | r, mAb | | Cell Signaling | |  |  |
| STF-1 (D1Z2A) XP® | #12800 | AB_2798030 | 1:500 | r, mAb | | Cell Signaling | |  |  |
| Vimentin (EPR3776) | ab92547 | AB_10562134 | 1:3,000 | r, mAb | | Abcam | |  |  |
| α smooth muscle actin antibody [EPR5368] | ab124964 | AB_11129103 | 1:1,000 | r, mAb | | Abcam | |  |  |
| α Tubulin  (B-7) | sc-5286 | AB_628411 | 1:1,000 | m, mAb | | Santa Cruz Biotech. | |  |  |
| β-actin | A5441 | AB_476744 | 1:10,000 | | m, mAb | | Sigma-Aldrich | |  |
| **Secondary antibodies** | | | | | | | | |  |
| goat anti-mouse IgG  (H+L), HRP | 31430 | AB_228307 | 1:5,000 | | g | | Thermo Fisher Scientific, Waltham, MA, USA | |  |
| goat anti-rabbit IgG  (H+L), HRP | 31460 | AB_228341 | 1:5,000 | | g | | Thermo Fisher Scientific | |  |
| Abbreviations used are: pAb, polyclonal antibody; mAb: monoclonal antibody; g, goat; m, mouse; r, rabbit. ^1^Data were taken from the Research Resource Identifier (RRID) portal, which is available at https://www.rrids.org/. * Distribution of this antibody has been discontinued. | | | | | | | | | |
