# Supplementary material for: Molecular characterization of the murine Leydig cell lines TM3 and MLTC-1
Source: Front Endocrinol (Lausanne). 2025 Dec 16;16:1715307. doi: 10.3389/fendo.2025.1715307 (PMC12747838; doi:10.3389/fendo.2025.1715307)
Supplement: Supplementary Table 2 — Primers used in this study. [file Table2.docx]

**Supplementary Table 2: Primers used in this study**

| **Gene** | **Accession No.** | **Forward primer (5'->3')** | **Location** | **Reverse primer (5'->3')** | **Location** | **Amplicon size (bp)** | **Method** | **Reference** |
| --- | --- | --- | --- | --- | --- | --- | --- | --- |
| *Ar* | NM_013476.4 | gatggtatttgccatgggttg | 3197-3217 | ggctgtacatccgagacttgtg | 3312-3291 | 116 | RT-PCR, RT-qPCR | (1) |
| *Cyp11a1* | NM_019779.4 | cgcttttcctttgagtccatc | 688-708 | tctggaggcaggttgagcat | 836-817 | 149 | RT-PCR, RT-qPCR | (2) |
| *Cyp17a1* | NM_007809.3 | tctgggcactgcatcacg | 1341-1358 | gctccgaagggcaaataact | 1464-1445 | 124 | RT-PCR, RT-qPCR | (3) |
| *Cyp19a1* | NM_007810.4 | acacatcatgctggacacct | 918-937 | tcaggtctccacgtctctca | 1216-1197 | 299 | RT-PCR, RT-qPCR | NCBI design tool |
| *Hsd17b3* | NM_008291.3 | cagcttccaaggcttttgtg | 657-676 | acaaactcatcggcggtctt | 813-794 | 157 | RT-PCR, RT-qPCR | (4) |
| *Hsd3b1* | NM_008293.4 | gtattccgaccagaaaccaagg | 487-508 | ggcacacttgcttgaacacag | 728-708 | 242 | RT-PCR, RT-qPCR | (2) |
| *Hsd3b6* | NM_013821.3 | atccacactgcagctgtcattg | 562-583 | atgatgctcttcctcgttgc | 765-746 | 204 | RT-PCR, RT-qPCR | (4) |
| *Insl3* | NM_013564.7 | tgctcctggctctggggtcc | 29-48 | cactgcagcagctcccggtc | 197-178 | 169 | RT-PCR, RT-qPCR | (4) |
| *Lhcgr* | NM_013582.3 | aatgggacgacgctaatctcgc | 638-659 | tgagcgtctgaatggactccag | 800-779 | 163 | RT-PCR,  RT-qPCR | (2) |
| *Lipe* | NM_010719.5 | agcgctggaggagtgtttt | 1680-1698 | ccgctctccagttgaacc | 1755-1738 | 76 | RT-qPCR | (5) |
| *Mgll* | NM_001166251.2 | tcggaacaagtcggaggt | 1053-1070 | tcagcagctgtatgccaaag | 1141-1122 | 89 | RT-qPCR | (6) |
| *Plin1* | NM_001113471.1 | aacgtggtagacactgtggtaca | 1232-1254 | tctcggaattcgctctcg | 1308-1291 | 77 | RT-qPCR | (7) |
| *Plin2* | NM_007408.4 | ctccactccactgtccacct | 827-846 | gcttatcctgagcaccctga | 911-892 | 85 | RT-qPCR | (8) |
| *Plin3* | NM_025836.3 | ccacaggatgctgaaaagg | 1012-1030 | tgatgtccctgaacatgctg | 1087-1068 | 76 | RT-qPCR | (8) |
| *Plin4* | NM_020568.3 | ggacttacaaacagcaacagacc | 245-267 | tctgtgagttggtggacacttt | 315-294 | 71 | RT-qPCR | (8) |
| *Plin5* | NM_025874.3 | gtccggtgatcagacagctc | 166-185 | tcgattcaccacattctgct | 238-219 | 73 | RT-qPCR | (8) |
| *Pnpla2* | NM_001163689.1 | tgaccatctgccttccaga | 1263-1281 | tgtaggtggcgcaagaca | 1351-1334 | 89 | RT-qPCR | (5) |
| *Ppia* | NM_008907.2 | tgtgccagggtggtgacttt | 226-245 | cgtttgtgtttggtccagcat | 369-349 | 144 | RT-qPCR | (9) |
| *Rn18s* | NR_003278.3 | ctcaacacgggaaacctcac | 1247-1266 | cgctccaccaactaagaacg | 1356-1341 | 110 | RT-PCR | (10) |
| *Rpl13a* | NM_009438.5 | taccagaaagtttgcttacctggg | 424-447 | tgcctgtttccgtaacctcaag | 574-553 | 151 | RT-PCR | (11) |
| *Rps6* | NM_009096.3 | cccatgaagcaaggtgttct | 225-244 | acaatgcatccacgaacaga | 346-327 | 122 | RT-qPCR | (12) |
| *Star* | NM_011485.5 | gaggttccacctgtgtgctg | 670-689 | caggtggttggcgaactcta | 905-886 | 236 | RT-PCR, RT-qPCR | (13) |

**References**

1. **Keil KP, Abler LL, Laporta J, Altmann HM, Yang B, Jarrard DF, Hernandez LL, Vezina CM**. Androgen receptor DNA methylation regulates the timing and androgen sensitivity of mouse prostate ductal development. Dev Biol. 2014;396:237-45. doi: 10.1016/j.ydbio.2014.10.006

2. **Duan N, Ran Y, Wang H, Luo Y, Gao Z, Lu X, Cui F, Chen Q, Xue B, Liu X**. Mouse testicular macrophages can independently produce testosterone and are regulated by Cebpb. Biological research. 2024;57:64. doi: 10.1186/s40659-024-00544-8

3. **Xu B, Gao L, Cui Y, Gao L, Dai X, Li M, Zhang Y, Ma X, Diao F, Liu J**. SET protein up-regulated testosterone production in the cultured preantral follicles. Reprod Biol Endocrinol. 2013;11:9. doi: 10.1186/1477-7827-11-9

4. **Hazra R, Jimenez M, Desai R, Handelsman DJ, Allan CM**. Sertoli cell androgen receptor expression regulates temporal fetal and adult Leydig cell differentiation, function, and population size. Endocrinology. 2013;154:3410-22. doi: 10.1210/en.2012-2273

5. **Sekine S, Yao A, Hattori K, Sugawara S, Naguro I, Koike M, Uchiyama Y, Takeda K, Ichijo H**. The Ablation of Mitochondrial Protein Phosphatase Pgam5 Confers Resistance Against Metabolic Stress. EBioMedicine. 2016;5:82-92. doi: 10.1016/j.ebiom.2016.01.031

6. **Iizasa S, Nagao K, Tsuge K, Nagano Y, Yanagita T**. Identification of genes regulated by lipids from seaweed Susabinori (Pyropia yezoensis) involved in the improvement of hepatic steatosis: Insights from RNA-Seq analysis in obese db/db mice. PLOS ONE. 2023;18:e0295591. doi: 10.1371/journal.pone.0295591

7. **De Siqueira MK, Li G, Zhao Y, Wang S, Ahn IS, Tamboline M, Hildreth AD, Larios J, Schcolnik-Cabrera A, Nouhi Z, Zhang Z, Tol MJ, Pandey V, Xu S, O'Sullivan TE, Mack JJ, Tontonoz P, Sallam T, Wohlschlegel JA, Hulea L, Xiao X, Yang X, Villanueva CJ**. PPARgamma-dependent remodeling of translational machinery in adipose progenitors is impaired in obesity. Cell Rep. 2024;43:114945. doi: 10.1016/j.celrep.2024.114945

8. **Asimakopoulou A, Vucur M, Luedde T, Schneiders S, Kalampoka S, Weiss TS, Weiskirchen R**. Perilipin 5 and Lipocalin 2 Expression in Hepatocellular Carcinoma. Cancers. 2019;11. doi: 10.3390/cancers11030385

9. **Mi J, Hooker E, Balog S, Zeng H, Johnson DT, He Y, Yu EJ, Wu H, Le V, Lee DH, Aldahl J, Gonzalgo ML, Sun Z**. Activation of hepatocyte growth factor/MET signaling initiates oncogenic transformation and enhances tumor aggressiveness in the murine prostate. The Journal of biological chemistry. 2018;293:20123-36. doi: 10.1074/jbc.RA118.005395

10. **Lin P, Lan X, Chen F, Yang Y, Jin Y, Wang A**. Reference gene selection for real-time quantitative PCR analysis of the mouse uterus in the peri-implantation period. PLoS One. 2013;8:e62462. doi: 10.1371/journal.pone.0062462

11. **Ganchala D, Pinto-Benito D, Baides E, Ruiz-Palmero I, Grassi D, Arevalo MA**. Kif21B mediates the effect of estradiol on the morphological plasticity of mouse hippocampal neurons. Front Mol Neurosci. 2023;16:1143024. doi: 10.3389/fnmol.2023.1143024

12. **Boaru SG, Borkham-Kamphorst E, Tihaa L, Haas U, Weiskirchen R**. Expression analysis of inflammasomes in experimental models of inflammatory and fibrotic liver disease. J Inflamm. 2012;9:49. doi: 10.1186/1476-9255-9-49

13. **Oh YS, Koh IK, Choi B, Gye MC**. ESR1 inhibits hCG-induced steroidogenesis and proliferation of progenitor Leydig cells in mice. Sci Rep. 2017;7:43459. doi: 10.1038/srep43459
