# Supplementary material for: Molecular characterization of the murine Leydig cell lines TM3 and MLTC-1
Source: Front Endocrinol (Lausanne). 2025 Dec 16;16:1715307. doi: 10.3389/fendo.2025.1715307 (PMC12747838; doi:10.3389/fendo.2025.1715307)
Supplement: Supplementary Table 4 — Short tandem repeat (STR) profiling of TM3 and MLTC-1 cells. [file Table4.docx]

**Supplementary Table 4: Short tandem repeat (STR) profiling of TM3 and MLTC-1 cells**

| **Cell line/entry** | **STR Marker** | | | | | | | | | | | | | | | | | | |
| --- | --- | --- | --- | --- | --- | --- | --- | --- | --- | --- | --- | --- | --- | --- | --- | --- | --- | --- | --- |
|  | **1-1** | **1-2** | **2-1** | **3-2** | **4-2** | **5-5** | **6-4** | **6-7** | **7-1** | **8-1** | **9-2** | **11-2** | **12-1** | **13-1** | **15-3** | **17-2** | **18-3** | **19-2** | **X-1** |
| TM3 | 15,16 | 15,17 | 9,16 | 13,14 | 19.3 | 11,12 | 16.3,18 | 15 | 25.2,26.2 | 13,16,17 | 15 | 15,18 | 16,17 | 16.2 | 20.3,22.3 | 15,16 | 18 | 9 | 23,24 |
| YC-D021* | 16 | 15,17 | 9,16 | 13,14 | 19.3 | 11,12 | 16.3;18 | 15 | 25.2,26.2 | 13,16,17 |  | 15,18 | 16 | 16.2 | 20.3,22.3 | 15,16 | 18 | 9 | 24 |
| CVCL_4326** | NA | NA | NA | NA | 19.3 | 12 | 16.3,17.3 | 15 | NA | NA | NA | NA | 16,17 |  | 20.3,22.3 | NA | 18 |  | 24 |
| **Cell line/ entry** | **STR Marker** | | | | | | | | | | | | | | | | | | |
|  | **1-1** | **1-2** | **2-1** | **3-2** | **4-2** | **5-5** | **6-4** | **6-7** | **7-1** | **8-1** | **9-2** | **11-2** | **12-1** | **13-1** | **15-3** | **17-2** | **18-3** | **19-2** | **X-1** |
| MLTC-1 | 16 | 19,20 | 16 | 14,15 | 20.3,21.3 | 17 | 18 | 16 | 26.2 | 16 | 18 | 16 | 17 | 17 | 22.3 | 15 | 16 | 13 | 28 |
| CVCL_3544*** | 16 | 19,20 | 16 | 14,15 | 20.3,21.3 | 17 | 18 | 16 | 26.2 | 16 | NA | 16 | 17 | 17 | 22.3 | 15 | 16 | 13 | 28 |

* STR data taken from information provided by ubigene for cell line TM3 (1). ** Data taken from ATCC database for entry CRL-2065 (2); *** Data taken from Cellosaurus data base entry CVCL_3544 that contained data taken from (3).

**References cited**

1. Ubigene. Available at: https://www.ubigene.com/instruction/product/TM3-%E7%BB%86%E8%83%9E%E4%BD%BF%E7%94%A8%E8%AF%B4%E6%98%8E%E4%B9%A6.pdf (last accessed 23 September 2025)
2. Cellosaurus TM3 (Mouse testis). Available at: https://www.cellosaurus.org/CVCL_4326 (last accessed 23 September 2025)
3. Almeida JL, Dakic A, Kindig K, Kone M, Letham DLD, Langdon S, Peat R, Holding-Pillai J, Hall EM, Ladd M, Shaffer MD, Berg H, Li J, Wigger G, Lund S, Steffen CR, Fransway BB, Geraghty B, Natoli M, Bauer B, Gollin SM, Lewis DW, Reid Y. Interlaboratory study to validate a STR profiling method for intraspecies identification of mouse cell lines. PLoS One. 2019 Jun 20;14(6):e0218412. doi: 10.1371/journal.pone.0218412
