# Supplementary figures and images for: Molecular characterization of the murine Leydig cell lines TM3 and MLTC-1
Source: Front Endocrinol (Lausanne). 2025 Dec 16;16:1715307. doi: 10.3389/fendo.2025.1715307 (PMC12747838; doi:10.3389/fendo.2025.1715307)

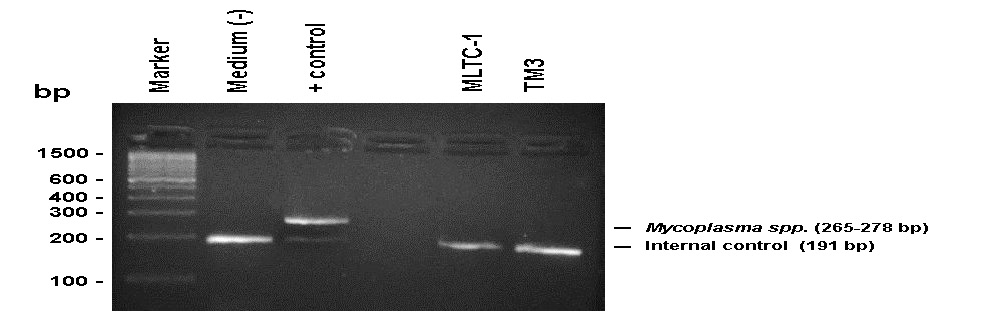

Supplement: Supplementary Figure 1 — Mycoplasma testing. [file Image1.jpeg]

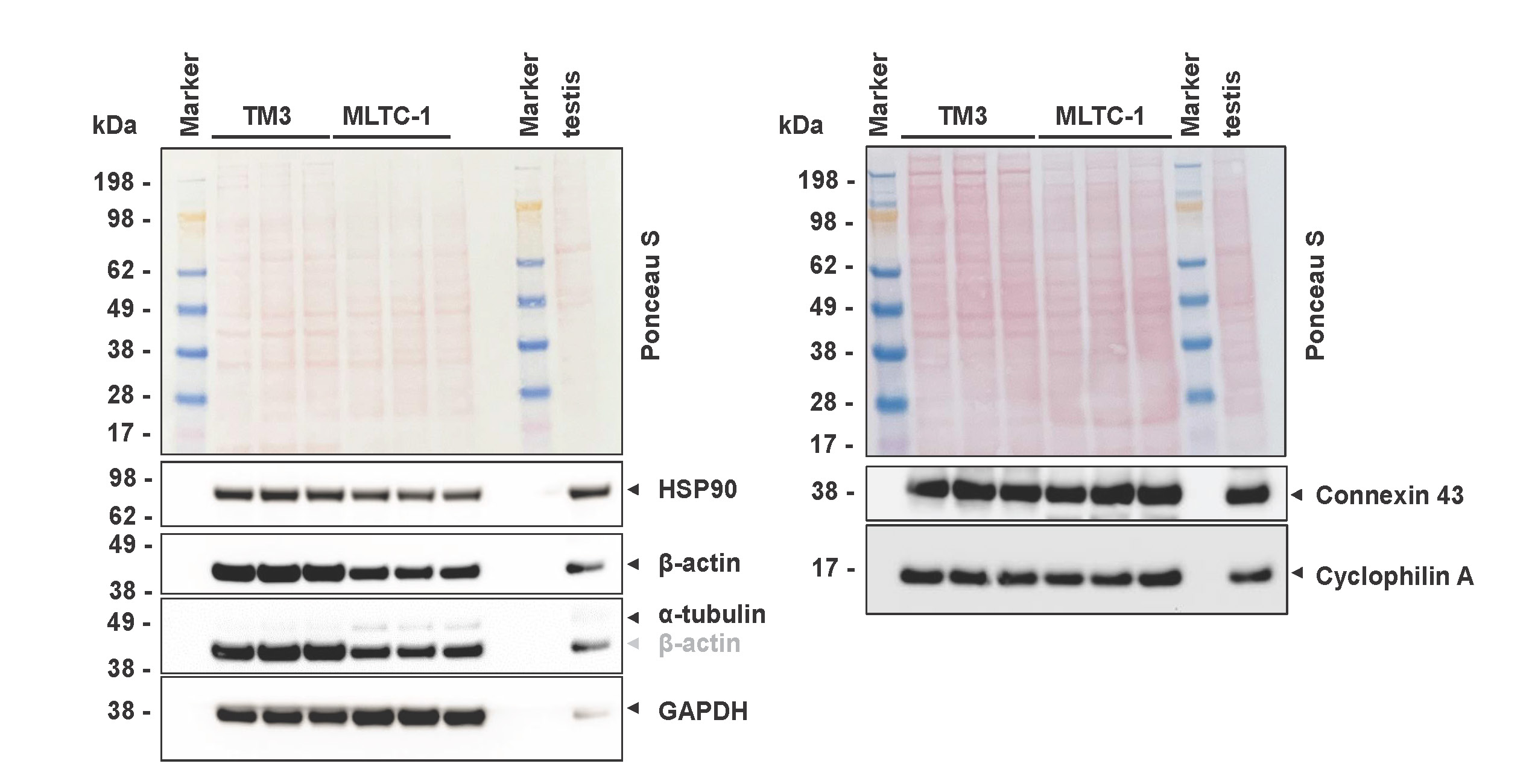

Supplement: Supplementary Figure 2 — Loading controls for Western blot analysis in TM3 and MLTC-1 cells. [file Image2.jpeg]

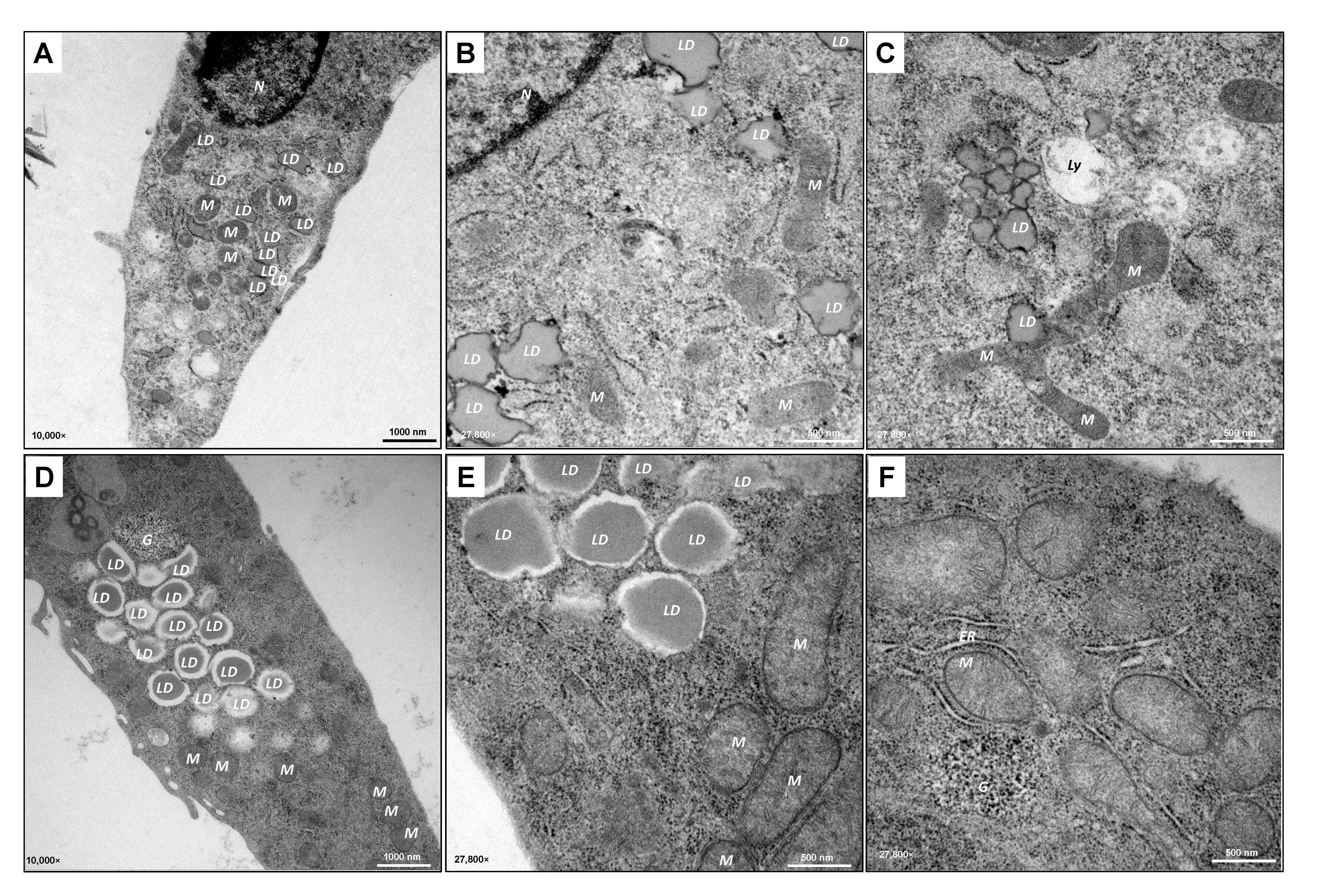

Supplement: Supplementary Figure 3 — Ultrastructural analysis of LC lines stimulated with oleic acid (OA). [file Image3.jpeg]

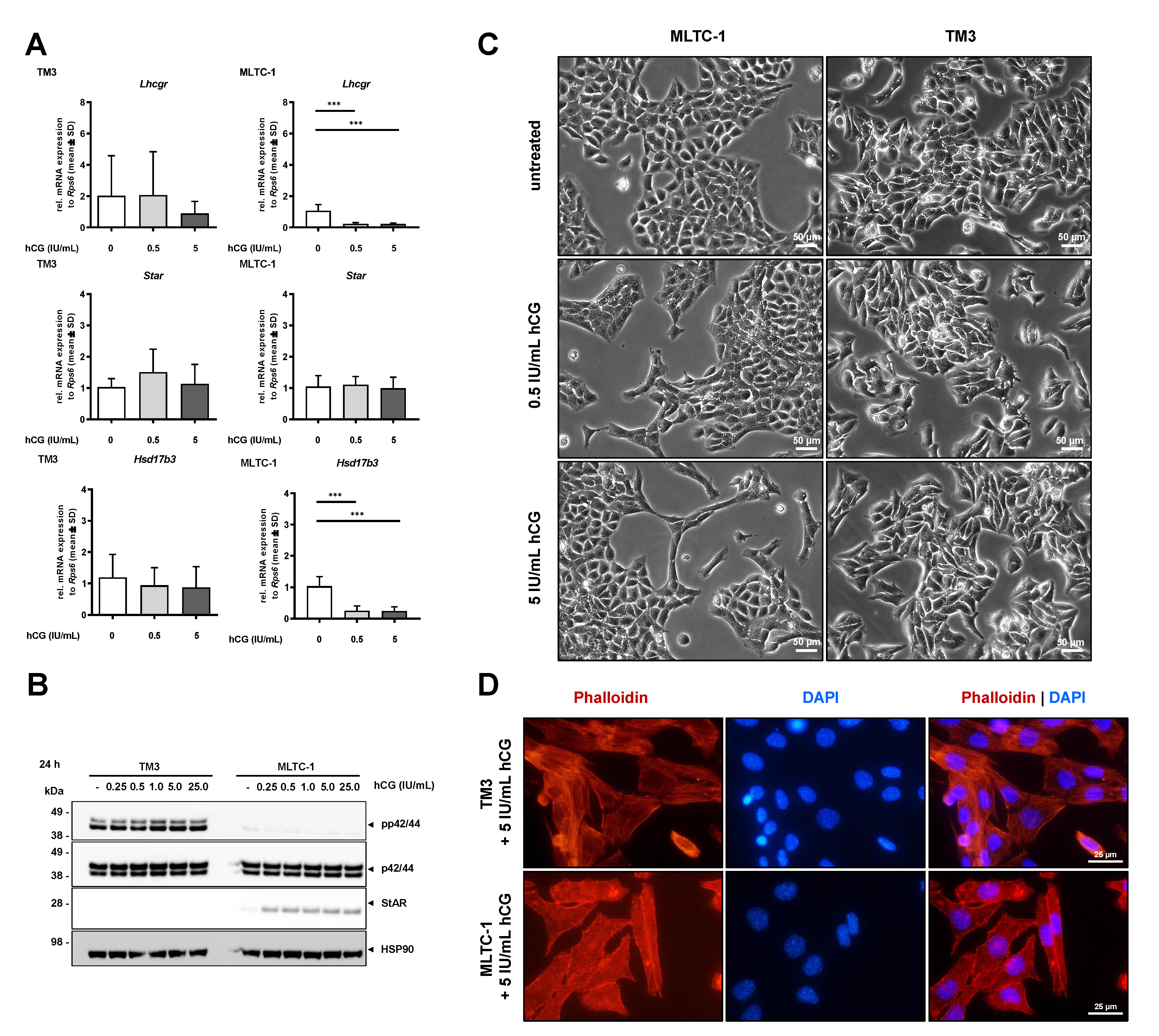

Supplement: Supplementary Figure 5 — Effects of chorionic gonadotropin (hCG) stimulation in TM-3 and MLTC-1 Leydig cells. [file Image5.jpeg]
